# Genome-wide association and genotype by environment interactions for growth traits in U.S. Red Angus cattle

**DOI:** 10.1186/s12864-022-08667-6

**Published:** 2022-07-16

**Authors:** Johanna L. Smith, Miranda L. Wilson, Sara M. Nilson, Troy N. Rowan, Robert D. Schnabel, Jared E. Decker, Christopher M. Seabury

**Affiliations:** 1 Department of Veterinary Pathobiology, Texas A&M University, College Station, 77843 USA; 2 Division of Animal Sciences, University of Missouri, Columbia, 65211 USA; 3 Genetics Area Program, University of Missouri, Columbia, 65211 USA; 4 Informatics Institute, University of Missouri, Columbia, 65211 USA

**Keywords:** GWAA, QTL, Genotype-by-environment interaction, Growth traits, Red Angus

## Abstract

**Background:**

Genotypic information produced from single nucleotide polymorphism (SNP) arrays has routinely been used to identify genomic regions associated with complex traits in beef and dairy cattle. Herein, we assembled a dataset consisting of 15,815 Red Angus beef cattle distributed across the continental U.S. and a union set of 836,118 imputed SNPs to conduct genome-wide association analyses (GWAA) for growth traits using univariate linear mixed models (LMM); including birth weight, weaning weight, and yearling weight. Genomic relationship matrix heritability estimates were produced for all growth traits, and genotype-by-environment (GxE) interactions were investigated.

**Results:**

Moderate to high heritabilities with small standard errors were estimated for birth weight (0.51 ± 0.01), weaning weight (0.25 ± 0.01), and yearling weight (0.42 ± 0.01). GWAA revealed 12 pleiotropic QTL (BTA6, BTA14, BTA20) influencing Red Angus birth weight, weaning weight, and yearling weight which met a nominal significance threshold (*P ≤* 1e-05) for polygenic traits using 836K imputed SNPs. Moreover, positional candidate genes associated with Red Angus growth traits in this study (i.e., *LCORL, LOC782905, NCAPG, HERC6, FAM184B, SLIT2, MMRN1, KCNIP4, CCSER1, GRID2, ARRDC3, PLAG1, IMPAD1, NSMAF, PENK, LOC112449660, MOS, SH3PXD2B, STC2, CPEB4*) were also previously associated with feed efficiency, growth, and carcass traits in beef cattle. Collectively, 14 significant GxE interactions were also detected, but were less consistent among the investigated traits at a nominal significance threshold (*P ≤* 1e-05); with one pleiotropic GxE interaction detected on BTA28 (24 Mb) for Red Angus weaning weight and yearling weight.

**Conclusions:**

Sixteen well-supported QTL regions detected from the GWAA and GxE GWAA for growth traits (birth weight, weaning weight, yearling weight) in U.S. Red Angus cattle were found to be pleiotropic. Twelve of these pleiotropic QTL were also identified in previous studies focusing on feed efficiency and growth traits in multiple beef breeds and/or their composites. In agreement with other beef cattle GxE studies our results implicate the role of vasodilation, metabolism, and the nervous system in the genetic sensitivity to environmental stress.

**Supplementary Information:**

The online version contains supplementary material available at 10.1186/s12864-022-08667-6.

## Background

Implementation of genomic selection methods into breeding programs has catalyzed production profitability within the beef cattle industry [1]. In addition to carcass and reproductive traits, the most commonly recorded traits for use in modern breeding programs are growth traits, such as birth weight, weaning weight, and yearling weight. However, genomic selection on these traits should consider that low and high estimated breeding values (EBVs) for birth weight have been found to be associated with reduced calf viability, and increased rates of dystocia events and perinatal mortality, respectively [2, 3]. Therefore, while birth weight has been considered a production indicator and treated as a selection criterion to increase calf viability as well as other economically important traits, modern beef breeding programs and production systems generally strive to increase calving ease while also maximizing both weaning weight and yearling weight [1, 3–5].

For at least two decades, studies have sought to identify quantitative trait loci (QTL) influencing bovine growth, body weight, and aspects of stature, including both linkage and modern genome-wide association analyses (GWAA); thereby underscoring the longstanding economic importance of efficient beef cattle production worldwide [6–12]. Moreover, QTL studies and modern genomic selection programs for economically important traits have been directly enabled by the generation of the bovine genome assembly, development of the Illumina Bovine SNP50 and 778K SNP arrays, and more recently, the demonstrated ability to accurately impute high-density genotypes, thereby enabling high-resolution analyses without the increased costs associated with direct genotyping [13–20]. Notably, several recent studies have established moderate heritability estimates for birth weight, weaning weight, and yearling weight in U.S. Gelbvieh, Angus, Limousin, Simmental, Hereford, and Red Angus beef cattle [20–25]. These studies also produce evidence for several relevant QTL and positional candidate genes; including orthologous genes *LCORL* and *PLAG1* that affect both human and bovine height as well as pleiotropic QTL influencing feed efficiency, growth traits, and carcass traits across multiple U.S. beef breeds [6, 10, 12, 20, 26–31]. However, the movement of germplasm (animals, semen, and embryos) across the U.S. in conjunction with the lack of tools to select for resilience to abiotic and biotic stressors has likely led to the loss of local adaptation in beef cattle [32]. Understanding genotype-by-environment interactions will allow us to identify the genes and biological processes involved in local adaptation. Genotype-by-environment (GxE) GWAA have been used alongside GWAA with the intent of identifying GxE interactions with complex traits [20, 33–35]. GxE GWAA are important to the beef industry as they identify individual ecoregions that could benefit from genomic selection [20, 33, 34].

The objective of this study was to identify loci with direct and genotype-by-environment effects on growth traits. Herein, we used 15,815 geographically diverse U.S. Red Angus beef cattle in conjunction with a union set of 836,118 (836K) imputed SNP variants to conduct GWAA and produce marker-based heritability estimates for birth weight, weaning weight, and yearling weight. Additionally, using thirty-year climate data and K-means clustering to assign all Red Angus beef cattle to discrete U.S. climate ecoregions, we estimated the significance of GxE interactions for birth weight, weaning weight, and yearling weight [32]. The present study represents the largest, high-density, single breed report to date that includes both standard GWAA and GxE GWAA for birth weight, weaning weight, and yearling weight; which was facilitated by an industry-supported research framework that includes accurate imputation to high-density genotypes for large-sample analyses [14, 19, 20]. The results of this study are expected to aid existing beef breeding programs and production systems by identifying QTL that may be included in future genotyping assays and genomic selection programs.

## Results and discussion

### Heritability estimates for growth traits in U.S. Red Angus beef cattle

Marker-based heritability estimates (i.e., chip heritability) were produced for birth weight, weaning weight, and yearling weight using standardized relatedness matrices (*G*_*S*_) with variance component analyses. Collectively, moderate to high heritability estimates with small standard errors (SE) were estimated for birth weight (0.51 ± 0.01), weaning weight (0.25 ± 0.01), and yearling weight (0.42 ± 0.01), respectively (Table 1). Moreover, these moderate to high heritability estimates for birth weight and weaning weight are similar to those produced by another study conducted on Red Angus cattle (0.58 ± 0.01 and 0.29 ± 0.01, respectively) [36]. Likewise, genetic correlations between traits were also high (birth weight and weaning weight = 0.54 ± 0.01; birth weight and yearling weight = 0.50 ± 0.01; weaning weight and yearling weight = 0.84 ± 0.01) (See Additional File 1).

**Table 1:** Variance component analysis with marker-based heritability estimates^a^

Trait	h^**2**^	SE^**b**^ of h^**2**^	V_**g**_^**c**^	V_**e**_^**d**^
**Birth Weight**	0.51	0.01	31.82	29.88
**Weaning Weight**	0.25	0.01	533.62	1546.23
**Yearling Weight**	0.42	0.01	2087.39	2820.06

^a^ h^2^ = V_g_ / (V_g_ + V_e_)

^b^ Standard error

^c^ Genetic variance component

^d^ Environmental variance component

### GWAA for birth weight, weaning weight, and yearling weight in U.S. Red Angus beef cattle

**Fig. 1:**
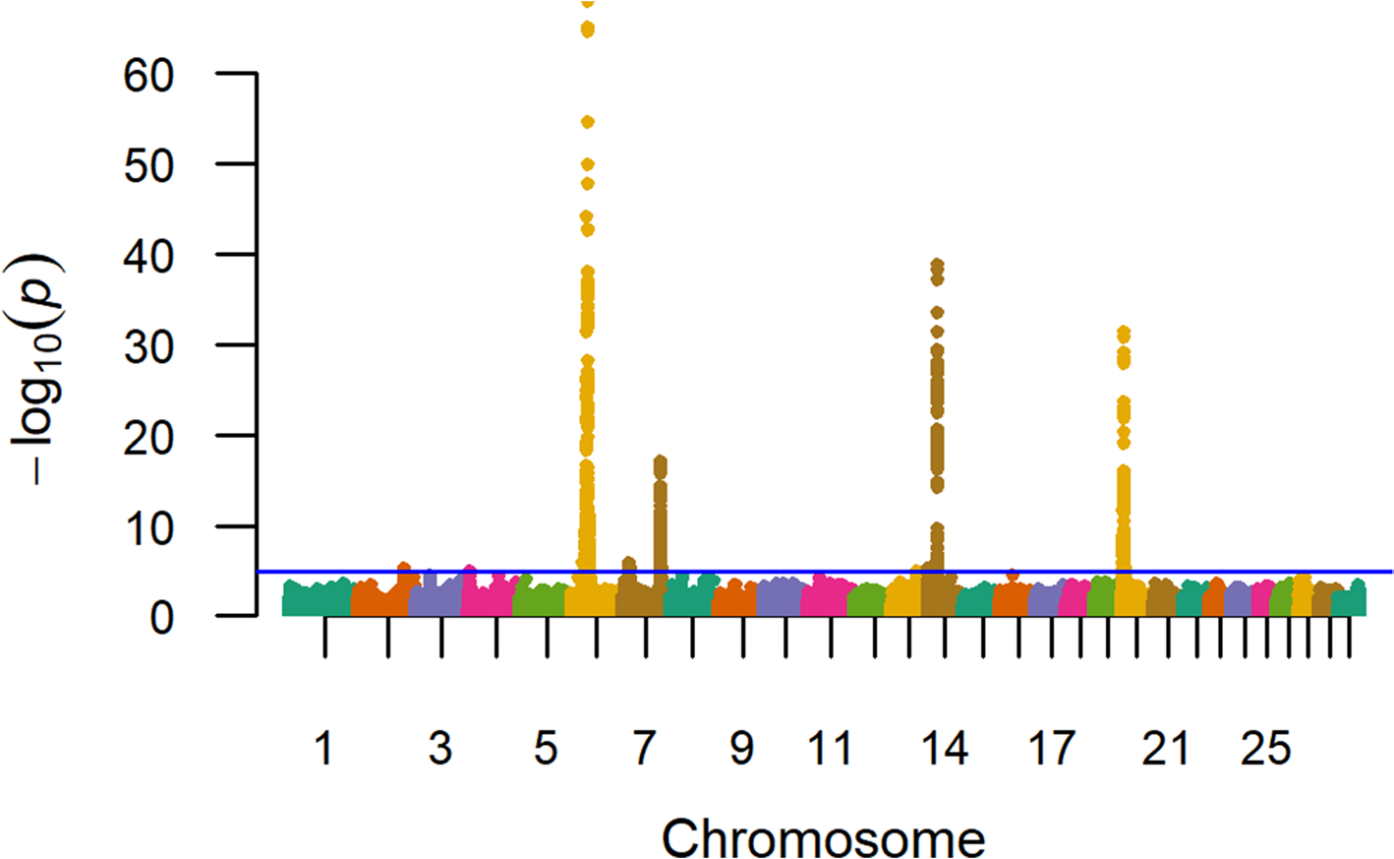
Birth weight QTL. Manhattan plot with -log_10_ *P*-values. Lead and supporting SNPs for QTL represented at or above the blue line (*P* ≤ 1e-05; −log_10_ *P*-values ≥ 5.00) for *n* = 15,815 U.S. Red Angus beef cattle. A summary of all markers passing the nominal significance threshold is presented in Table 2

The results of our 836K single-marker GWAA for birth weight are presented in Fig. 1; with detailed summary data for 19 QTL which met a nominal significance threshold for polygenic traits (*P ≤* 1e-05) described in Table 2 (Additional File 1) [71]. A comparison of birth weight QTL detected for U.S. Red Angus, Simmental, and Gelbvieh beef cattle as well as Holstein Jersey crossbred dairy cattle, revealed overlapping signals on BTA6, BTA14, and BTA20, suggesting that these birth weight QTL are not breed-specific, but rather, are likely to be more generally involved in bovine species growth processes (Table S1; Additional File 2) [9, 20, 72]. Positional candidate genes detected via birth weight GWAA revealed previous associations with aspects of cattle growth, development, feed efficiency, and carcass traits (i.e., *LCORL, LOC782905, NCAPG, PLAG1, LOC104975192, STC2, HERC6, LOC112449660, IMPAD1, SLIT2, LOC101905238, ARRDC3, LOC783392, CPEB4, MMRN1, SH3PXD2B, KCNIP4, GRID2, CCSER1, NSMAF, LOC107133116, ENC1, LOC112443028*), as well as milk production (*LOC101906669, TRNAG-UCC*), and immune response (*SLURP1*), as detailed in Table 2 [37–70]. Notably, all but two lead SNPs (i.e., 6_37 Mb, 6_35 Mb) were located in noncoding regions, which is concordant with recent studies of feed efficiency and growth traits in beef cattle (Table S1; Additional File 1, Additional File 2) [20, 31]. Additionally, a QTL was detected on BTA6 (42 Mb), but with less statistical support, and included the positional candidate genes *LOC782172* and *ADGRA3*; which have previously been associated with U.S. Gelbvieh growth traits (Table S2; Additional File 2) [20]. The genomic inflation factor for *P*-value estimates obtained from the birth weight GWAA are presented in Table S3 (Additional File 2).

**Table 2:** Summary of QTL detected for birth weight in U.S. Red Angus cattle

Chr_Mb^**a**^	-log_**10**_ P-value	Regression Beta	MAF^**b**^	Supporting SNPs^**c**^	Positional Candidate Genes	Lead SNP Location	Scientific Precedence [reference]; organism; trait
*6_38*	67.949	−3.123	0.283	102	*LCORL, LOC782905*	Intergenic	[20, 24, 26, 37–45]; Cattle; Birth, weaning and yearling weight, fat and protein percentage, carcass weight, fat thickness, dystocia, average daily gain and dry matter intake, hip height, ribeye area, calving ease, stature, feed intake and gain
*6_37*	47.734	−2.302	0.359	183	*NCAPG*	Exon^d^	[20, 24, 39, 40, 42–45]; Cattle; Weaning and yearling weight, calving ease, dystocia, average daily gain, dry matter intake, fetal growth, daily feed intake, muscle growth
*14_23*	38.772	2.800	0.110	64	*PLAG1*	3’UTR	[11, 20, 24, 26, 31, 46]; Cattle; Birth weight, mid-test metabolic weight, yearling and carcass weight, stature, milk protein yield, stature
*20_05*	31.330	1.815	0.436	100	*LOC104975192, STC2*	Intergenic	[20, 24, 31, 40, 42, 47, 48]; Cattle; Birth, weaning and carcass weight, average daily gain, dry matter intake, stature, mature rate, mid-test metabolic weight
*6_36*	26.156	−2.267	0.219	63	*HERC6*	Intron	[20, 40, 42, 49–51]; Cattle; Birth, weaning, and yearling weight, mature weight, average daily gain, dry matter intake, backfat thickness, conception rate, milk production traits, protein ubiquitination
*14_24*	25.908	2.143	0.113	80	*LOC112449660, IMPAD1*	Intergenic	[20, 24, 26, 29, 31, 46]; Cattle; Birth, weaning, and yearling weight, calving ease, carcass weight, milk and protein yield, stature, stillbirth, body size, mid-test metabolic weight
*6_39*	21.788	−1.569	0.270	35	*LOC782905, SLIT2*	Intergenic	[20, 24, 37, 40, 42, 52]; Cattle; Birth, weaning, and yearling weight, calving ease, carcass weight, average daily gain, dry matter intake, backfat thickness, ribeye area, hip height
*7_91*	17.095	1.339	0.263	70	*LOC101905238, ARRDC3*	Intergenic	[24, 30, 31, 46, 53]; Cattle; Milk protein yield, chest width and bone quality, calving ease, average daily gain, growth and muscularity, birth, weaning, and yearling weight, ribeye area
*20_06*	15.122	−1.372	0.382	12	*LOC783392, CPEB4*	Intergenic	[40, 48, 49, 54, 55]; Cattle; Fat percentage, dry matter intake, mature weight, conception rate, mRNA and protein expression in meiosis regulation
*6_35*	14.833	−1.305	0.317	32	*MMRN1*	Exon^e^	[24, 43, 56–58]; Cattle, human, sheep; Birth weight, dry matter intake, fetal growth, weaning weight, winter tolerance under metabolic stress response
*6_40*	14.409	2.002	0.060	47	*SLIT2*	Intron	[20, 24, 40, 42, 59, 60]; Cattle, mouse; Birth, weaning, and yearling weight, calving ease, average daily gain, carcass weight, hip height, milk fat and protein, development of central nervous system
*20_04*	11.639	−1.088	0.438	12	*SH3PXD2B*	Intron	[24, 40, 61]; Cattle, human; Birth and weaning weight, carcass weight, fat thickness, calving ease, average daily gain, development of eyes, heart, and bone
*6_41*	11.067	1.171	0.264	57	*KCNIP4*	Intron	[20, 24, 38, 40, 42, 62, 63]; Cattle, human; Birth, weaning, and yearling weight, protein percentage, average daily gain, ribeye area, carcass weight, milk fatty acid composition, potassium channel activity
*6_32*	9.335	0.985	0.325	57	*GRID2*	Intron	[40, 42, 46, 64]; Human, cattle; Mammalian nervous system mediation, dry matter intake, average daily gain, birth weight, milk fat yield
*6_34*	8.662	−1.095	0.285	14	*CCSER1*	Intron	[20, 49, 58, 65]; Cattle, human, sheep; Birth and weaning weight, conception rate, regulator of mitosis, feed intake
*14_25*	6.648	0.805	0.311	41	*NSMAF, LOC107133116*	Intergenic	[20, 24, 29, 31, 40, 47, 59, 66, 67]; Cattle, human; Birth, weaning, and yearling weights, calving ease, carcass weight, average daily gain, dry matter intake, hip height, stature, backfat thickness, ribeye area, lean meat yield, stillbirth, residual feed intake, immune system response
*20_07*	6.424	0.725	0.378	26	*ENC1, LOC112443028*	Intergenic	[49, 68, 69]; Cattle, mouse; Conception rate, residual feed intake, development of nervous system
*14_22*	5.827	−1.153	0.068	15	*LOC101906669, TRNAG-UCC*	Intergenic	[11, 40, 41]; Cattle; Average daily gain, hip height, protein percentage
*14_02*	5.239	0.762	0.124	5	*SLURP1*	3’UTR	[49, 54, 70]; Rat, cattle; Immune response, protein, fat and milk yield, fat and protein percentage, number of breedings until conception

^a^ Chromosome_Megabase

^b^ Minor Allele Frequency

^c^ Single Nucleotide Polymorphisms

^d^ Indicates a predicted nonsynonymous mutation Ile➔Met, exon 9

^e^ Indicates a predicted nonsynonymous mutation Gln➔His, exon 6

**Fig. 2:**
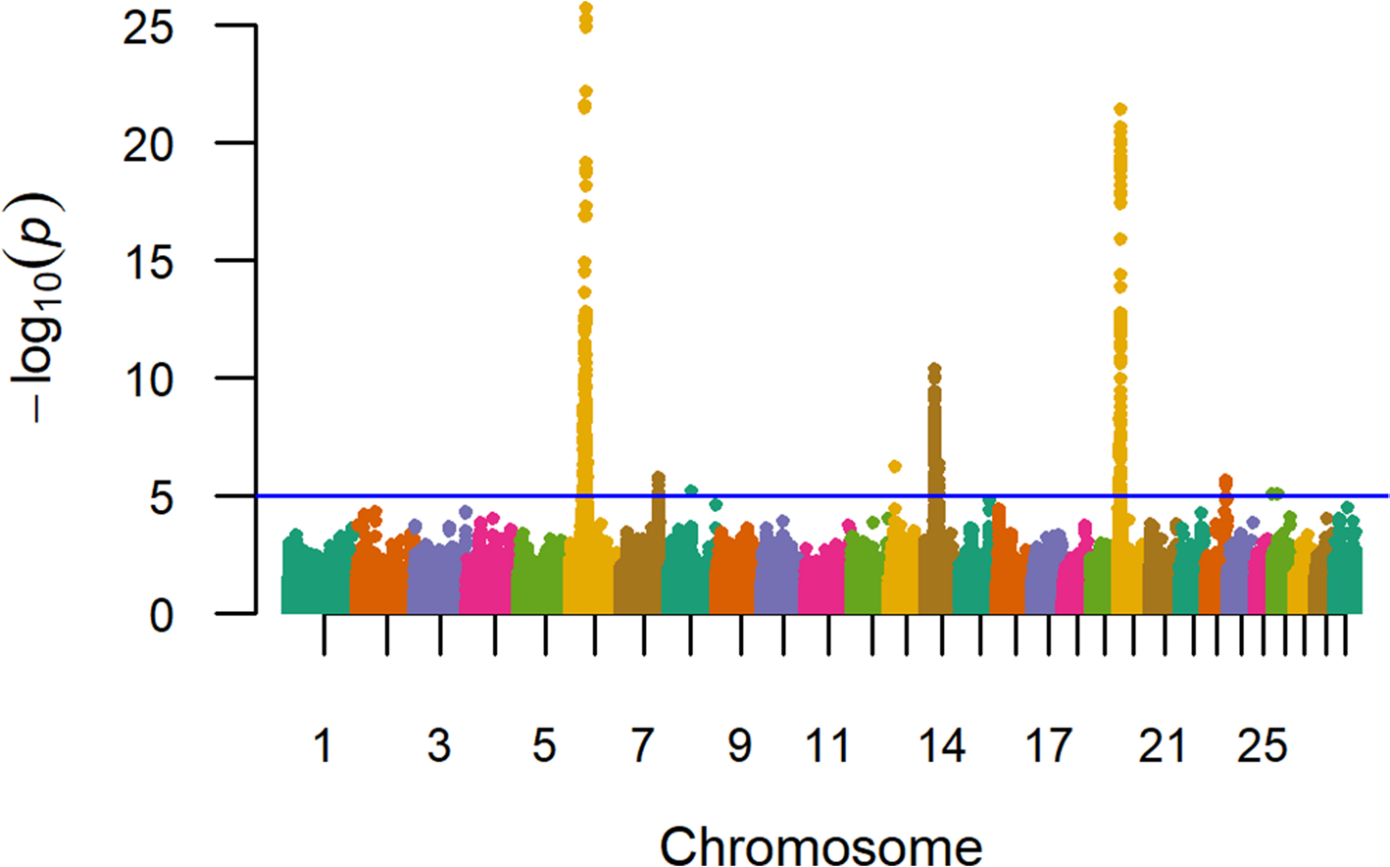
Weaning weight QTL. Manhattan plot with -log_10_ *P*-values. Lead and supporting SNPs for QTL represented at or above the blue line (*P* ≤ 1e-05; −log_10_ *P*-values ≥ 5.00) for *n* = 15,620 U.S. Red Angus beef cattle. A summary of all markers passing the nominal significance threshold is presented in Table 3

Single-marker GWAA (836K) for weaning weight in U.S. Red Angus beef cattle produced evidence for 14 QTL (*P ≤* 1e-05), as defined by their lead SNPs (Table 3, Fig. 2; Additional File 1). Similar to a recent analysis of U.S. Gelbvieh beef cattle [20], the weaning weight QTL regions detected for U.S. Red Angus cattle suggest extensive pleiotropy with birth weight, as would be expected due to high genetic correlations between the two traits [4]. This includes the shared positional candidate genes on BTA6 (*LCORL, LOC782905, HERC6, CCSER1, SLIT2, GRID2*), BTA14 (*LOC112449660*), and BTA20 (*LOC104975192, STC2, SH3PXD2B*) (Table 2, Table 3, Table S1; Additional File 2)*.* Additional positional candidate genes identified for weaning weight QTL include those associated with growth and development (*FAM184B, LOC112447052, NSG2, LOC112449630, MOS, PENK, MIR3660, CETN3*) (Table 3) [73–77]. All weaning weight QTL detected by GWAA were located in noncoding regions. An additional pleiotropic QTL was noted on BTA6 (42 Mb), but with less statistical support, which is the same QTL detected in the birth weight GWAA (Table S2, Table S4; Additional File 2). The genomic inflation factor for *P*-value estimates obtained from the weaning weight GWAA are displayed in Table S3 (Additional File 2).

**Table 3:** Summary of QTL detected for weaning weight in U.S. Red Angus cattle

Chr_Mb^**a**^	-log_**10**_ P-value	Regression Beta	MAF^**b**^	Supporting SNPs^**c**^	Positional Candidate Genes	Lead SNP Location	Scientific Precedence [reference]; organism; trait
*6_38*	25.669	−10.153	0.283	66	*LCORL, LOC782905*	Intergenic	[20, 24, 26, 37–45]; Cattle; Birth, weaning and yearling weight, fat and protein percentage, carcass weight, fat thickness, dystocia, average daily gain and dry matter intake, hip height, ribeye area, calving ease, stature, feed intake and gain
*6_37*	21.575	−8.352	0.328	121	*FAM184B*	Intron	[20, 24, 39, 40, 42]; Cattle; Weaning and yearling weights, calving ease, dystocia, average daily gain, dry matter intake, daily feed intake
*20_05*	21.392	−7.859	0.444	94	*LOC104975192, STC2*	Intergenic	[20, 24, 31, 40, 42, 47, 48]; Cattle; Birth, weaning and carcass weight, average daily gain, dry matter intake, stature, mature rate, mid-test metabolic weight
*6_36*	16.846	− 9.442	0.219	30	*HERC6*	Intron	[20, 40, 42, 49–51]; Cattle; Birth, weaning, and yearling weight, mature weight, average daily gain, dry matter intake, backfat thickness, conception rate, milk production traits, protein ubiquitination
*6_35*	11.962	−5.632	0.356	26	*LOC112447052*	Intron	[24, 40, 43, 56]; Cattle; Birth weight, dry matter intake, fetal growth, weaning weight
*20_06*	10.817	− 5.963	0.385	7	*NSG2*	3’UTR	[40, 48, 49, 54, 73]; Cattle, mouse; Fat percentage, dry matter intake, mature weight, conception rate, synapse formation and maintenance
*14_23*	10.334	7.667	0.110	47	*LOC112449630, MOS*	Intergenic	[11, 20, 24, 26, 46]; Cattle; Birth, yearling and carcass weights, milk protein and fat yield, stature
*6_34*	8.719	− 5.723	0.286	21	*CCSER1*	Intron	[20, 49, 58, 65]; Cattle, human, sheep; Birth and weaning weight, conception rate, regulator of mitosis, feed intake
*6_39*	8.370	−5.285	0.270	21	*LOC782905, SLIT2*	Intergenic	[20, 24, 37, 40, 42, 52]; Cattle; Birth, weaning, and yearling weight, calving ease, carcass weight, average daily gain, dry matter intake, backfat thickness, ribeye area, hip height
*20_04*	8.218	−4.810	0.438	12	*SH3PXD2B*	Intron	[24, 40, 61]; Cattle, human; Birth and weaning weights, carcass weight, fat thickness, calving ease, average daily gain, development of eyes, heart, and bone
*14_24*	6.670	6.144	0.099	31	*PENK, LOC112449660*	Intergenic	[20, 24, 26, 29, 31, 46, 74]; Cattle, rat; Birth, weaning, and yearling weights, calving ease, carcass weight, milk and protein yield, stillbirth, body size, mid-test metabolic weight, bone development
*6_40*	5.810	6.922	0.060	5	*SLIT2*	Intron	[20, 24, 40, 42, 59, 60]; Cattle, mouse; Birth, weaning, and yearling weight, calving ease, average daily gain, carcass weight, hip height, milk fat and protein, development of central nervous system
*7_90*	5.781	−5.278	0.097	5	*MIR3660, CETN3*	Intergenic	[42, 49, 75–77]; Cattle, human, mice; Birth and yearling weights, conception rate, cell proliferation control, insulin response, cell regulation, centriole duplication and mitosis in embryonic development
*6_32*	5.366	4.287	0.277	26	*GRID2*	Intron	[40, 42, 46, 64]; Human, cattle; Mammalian nervous system mediation, dry matter intake, average daily gain, birth weight, milk fat yield

^a^ Chromosome_Megabase

^b^ Minor Allele Frequency

^c^ Single Nucleotide Polymorphisms

**Fig. 3:**
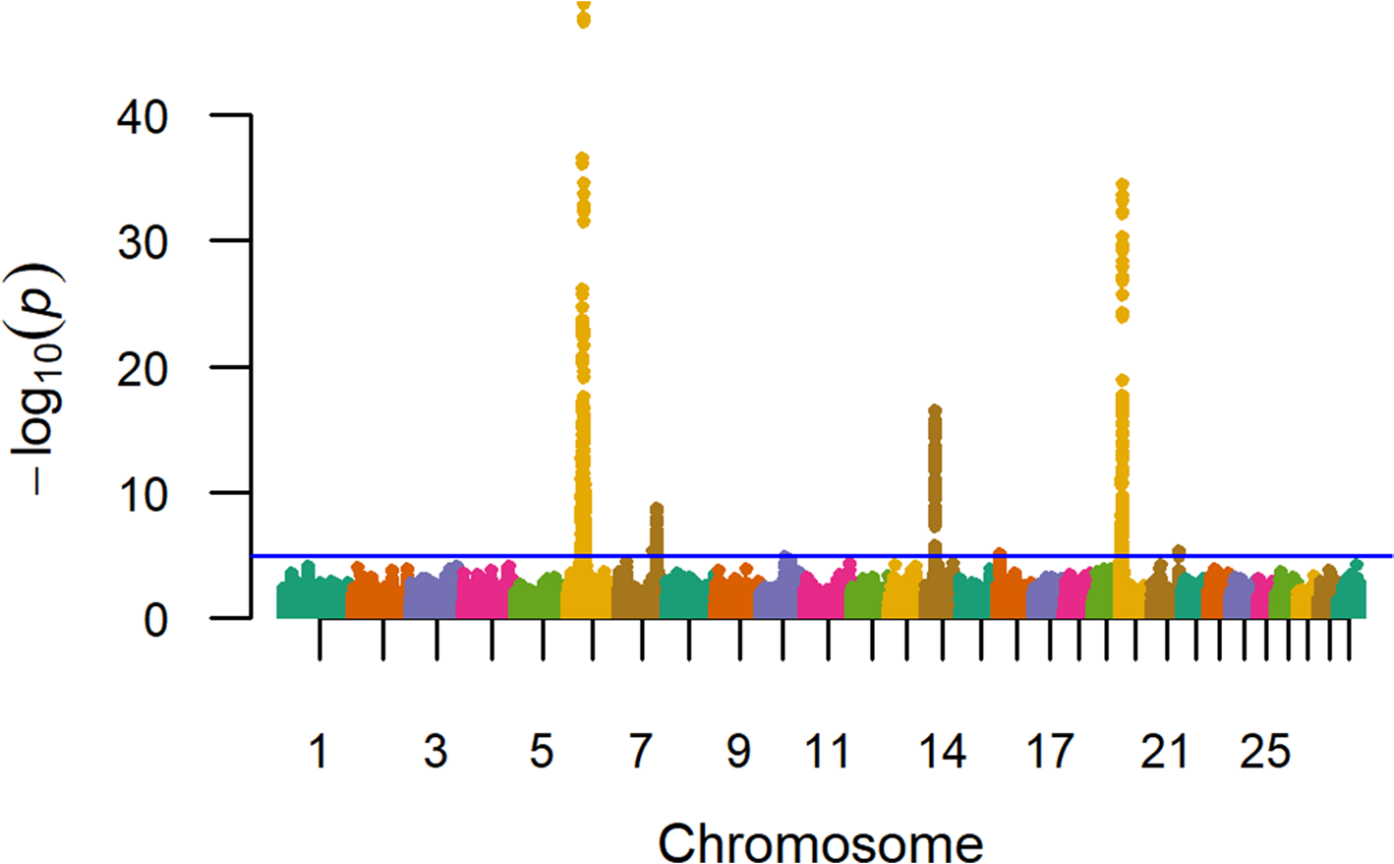
Yearling weight QTL. Manhattan plot with -log_10_ *P*-values. Lead and supporting SNPs for QTL represented at or above the blue line (*P* ≤ 1e-05; −log_10_ *P*-values ≥ 5.00) for *n* = 12,388 U.S. Red Angus beef cattle. A summary of all markers passing the nominal significance threshold is presented in Table 4

Similar to a recent study of U.S. Gelbvieh growth traits [20], our GWAA for yearling weight in U.S. Red Angus beef cattle also identified positional candidate genes shared across all three investigated traits on BTA6 (*LCORL, LOC782905, HERC6, SLIT2,* and *CCSER1*), BTA14 (*LOC112449660*), and BTA20 (*STC2* and *SH3PXD2B*) (Table 4, Fig. 3, Table S1; Additional File 1, Additional File 2). Evidence for pleiotropic QTL influencing birth weight (Table 2) and yearling weight (Table 4) was also noted via overlapping positional candidate genes for these traits on BTA6 (*KCNIP4, MMRN1*), BTA7 (*LOC101905238, ARRDC3, LOC112447488, LOC112447489*), BTA14 (*PLAG1*), and BTA20 (*LOC783392, CPEB4*) (Table S1; Additional File 2). Likewise, a comparison of U.S. Red Angus QTL detected for weaning weight (Table 3) and yearling weight (Table 4) also revealed evidence for pleiotropic QTL influencing these traits via shared positional candidate genes on BTA6 and BTA14, including *FAM184B* and *PENK,* respectively. Positional candidate genes for QTL on BTA7, BTA20, and BTA21 which were only detected for yearling weight have been associated with general growth and development in *Xenopus laevis* (*KCNIP1*) [78], as well as bovine milk production (*LOC112447488*) (Table 4, Fig. 3, Table S1; Additional File 2) [46]. Collectively, two of the 16 lead SNPs (20_05 Mb, *STC2*; 6_35 Mb, *MMRN1*) noted for yearling weight QTL were located within coding regions (Table 4). Interestingly, *STC2* has previously been associated with body size, feed efficiency, and growth in cattle [20, 24, 31, 40, 42, 47]; whereas *MMRN1* has been associated with growth, feed efficiency, and metabolic stability during weather stress in cattle (Table 4, Table S1; Additional File 1, Additional File 2) [24, 43, 56–58]. Despite less statistical support overall, the QTL on BTA6 at 42 Mb (i.e., *LOC782172* and *ADGRA3)* was detected for birth weight, weaning weight, and yearling weight in the Red Angus GWAA (Table S2, Table S4, Table S5; Additional File 2); as was the QTL on BTA6 at 32 Mb (Table 2, Table 3, Table S5; Additional File 2), and the QTL at BTA7 at 91 Mb (Table 2, Table 4, Table S5; Additional File 2). Finally, it should be noted that a pleiotropic QTL was also detected on BTA7 at 90 Mb for both weaning weight and yearling weight; albeit with less overall statistical support (Table 3, Table S5; Additional File 2). Genetic correlations estimated for all growth traits are summarized in Additional File 1. The genomic inflation factor for yearling weight GWAA is reported in Table S3 (Additional File 2). Genomic inflation factors (λ) larger than 1 are expected for well-powered studies of polygenic traits [79, 80], reflecting the large number of genomic loci influencing variation in these traits.

**Table 4:** Summary of QTL detected for yearling weight in U.S. Red Angus cattle

Chr_Mb^**a**^	-log_**10**_ P-value	Regression Beta	MAF^**b**^	Supporting SNPs^**c**^	Positional Candidate Genes	Lead SNP Location	Scientific Precedence [reference]; organism; trait
*6_38*	48.689	−24.991	0.282	87	*LCORL, LOC782905*	Intergenic	[20, 24, 26, 37–45]; Cattle; Birth, weaning and yearling weight, fat and protein percentage, carcass weight, fat thickness, dystocia, average daily gain and dry matter intake, hip height, ribeye area, calving ease, stature, feed intake and gain
*6_37*	36.517	−19.339	0.328	173	*FAM184B*	Intron	[20, 24, 39, 40, 42]; Cattle; Weaning and yearling weights, calving ease, dystocia, average daily gain, dry matter intake, daily feed intake
*20_05*	34.429	−18.105	0.441	116	*STC2*	Exon^d^	[20, 24, 31, 40, 42, 47, 48]; Cattle; Birth, weaning and carcass weight, average daily gain, dry matter intake, stature, mature rate, mid-test metabolic weight
*6_36*	25.670	−21.048	0.219	49	*HERC6*	Intron	[20, 40, 42, 49–51]; Cattle; Birth, weaning, and yearling weight, mature weight, average daily gain, dry matter intake, backfat thickness, conception rate, milk production traits, protein ubiquitination
*6_39*	19.104	−14.508	0.271	45	*LOC782905, SLIT2*	Intergenic	[20, 24, 39, 42, 44, 54]; Cattle; Birth, weaning, and yearling weight, calving ease, carcass weight, average daily gain, dry matter intake, backfat thickness, ribeye area, hip height
*14_23*	16.513	17.008	0.116	47	*PLAG1*	3’UTR	[11, 20, 24, 26, 31, 46]; Cattle; Birth weight, mid-test metabolic weight, yearling and carcass weight, stature, milk protein yield, stature
*20_06*	15.250	−12.790	0.390	24	*LOC783392, CPEB4*	Intergenic	[40, 48, 49, 54, 55]; Cattle; Fat percentage, dry matter intake, mature weight, conception rate, mRNA and protein expression in meiosis regulation
*6_40*	13.214	−11.340	0.276	69	*KCNIP4*	Intron	[20, 24, 42, 56, 59, 60, 63, 66]; Cattle, mouse, human; Birth, weaning, and yearling weights, calving ease, mature weight, average daily gain, carcass weight, hip height, post-weaning weight, liver weight, potassium channel function
*6_35*	12.635	−11.427	0.315	40	*MMRN1*	Exon^e^	[24, 43, 56–58]; Cattle, human, sheep; Birth weight, dry matter intake, fetal growth, weaning weight, winter tolerance under metabolic stress response
*6_34*	12.607	−12.734	0.286	36	*CCSER1*	Intron	[20, 49, 58, 65]; Cattle, human, sheep; Birth and weaning weight, conception rate, regulator of mitosis, feed intake
*14_24*	12.459	14.915	0.102	42	*PENK, LOC112449660*	Intergenic	[20, 24, 26, 29, 31, 46, 74]; Cattle, rat; Birth, weaning, and yearling weights, calving ease, carcass weight, milk and protein yield, stillbirth, body size, mid-test metabolic weight, bone development
*20_04*	10.984	−10.008	0.442	25	*SH3PXD2B*	Intron	[24, 40, 61]; Cattle, human; Birth and weaning weights, carcass weight, fat thickness, calving ease, average daily gain, development of eyes, heart, and bone
*6_41*	10.036	8.987	0.486	41	*KCNIP4*	Intron	[20, 24, 38, 40, 42, 62, 63]; Cattle, human; Birth, weaning, and yearling weight, protein percentage, average daily gain, ribeye area, carcass weight, milk fatty acid composition, potassium channel activity
*6_33*	9.693	−9.134	0.299	6	*CCSER1*	Intron	[24, 49, 65]; Cattle, human; Carcass weight, fat thickness, conception rate, regulator of mitosis
*7_91*	8.726	8.879	0.240	39	*LOC112447488, LOC112447489, LOC101905238, ARRDC3*	Intergenic	[24, 30, 31, 46, 53]; Cattle; Milk protein yield, chest width and bone quality, calving ease, average daily gain, growth and muscularity, birth, weaning, and yearling weight, ribeye area
*20_03*	8.002	7.171	0.472	6	*KCNIP1*	Intron	[78]; Frog, zebrafish; Development of neural plate, associated with cardiac myocytes

^a^ Chromosome_Megabase

^b^ Minor Allele Frequency

^c^ Single Nucleotide Polymorphisms

^d^ Indicates a predicted nonsynonymous mutation Pro➔Ala, exon 2

^e^ Indicates a predicted nonsynonymous mutation Gln➔His, exon 6

### GxE GWAA for birth weight, weaning weight, and yearling weight in U.S. Red Angus beef cattle

**Fig. 4:**
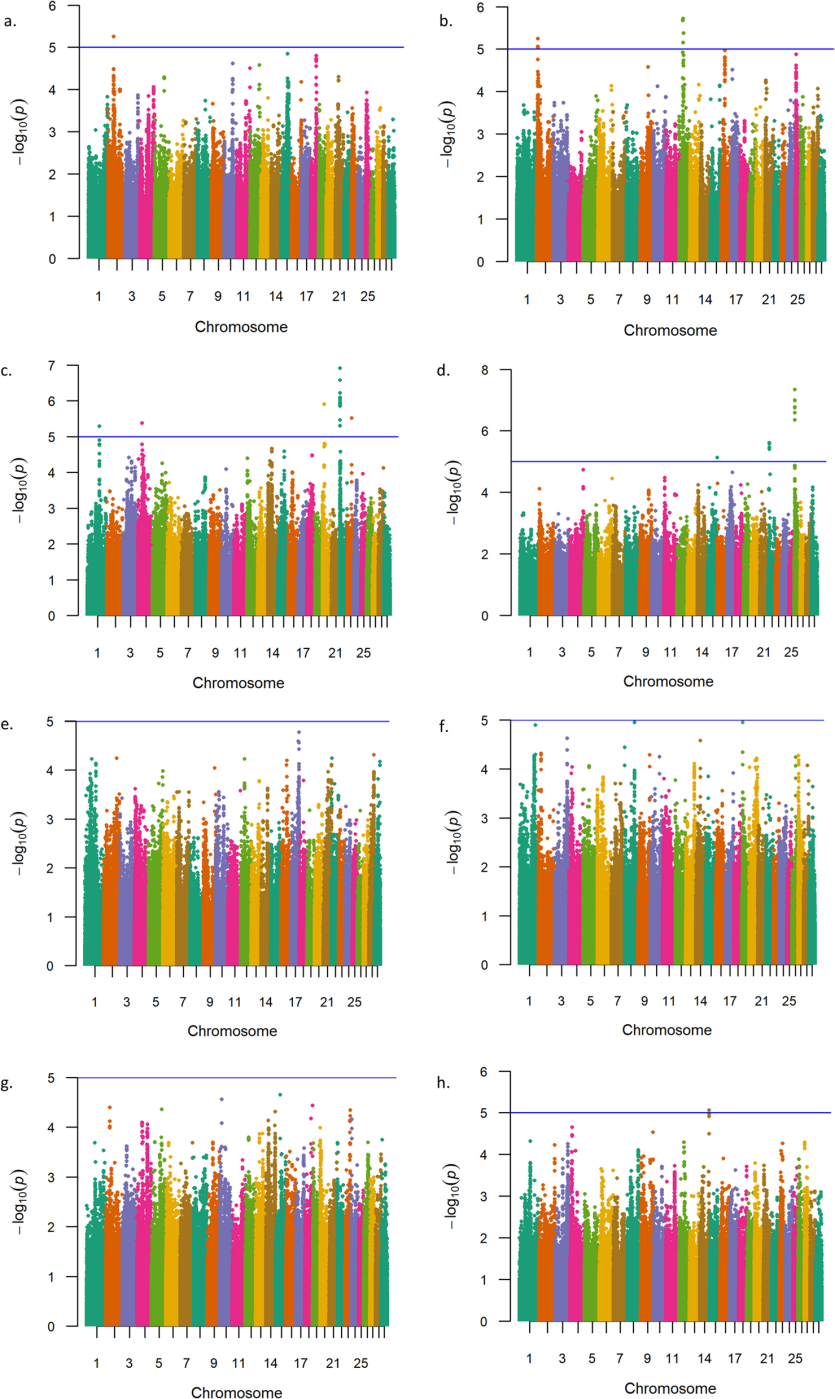
Birth weight genotype-by-environment interactions. Manhattan plots with -log_10_ *P*-values for U.S. Desert Ecoregion (**a**), U.S. Southeast Ecoregion (**b**), U.S. High Plains Ecoregion (**c**), U.S. Arid Prairie Ecoregion (**d**), U.S. Foothills Ecoregion (**e**), U.S. Forested Mountains Ecoregion (**f**), U.S. Fescue Belt Ecoregion (**g**), and U.S. Upper Midwest and Northeast Ecoregion (**h**). Lead and supporting SNPs for interactions represented at or above the blue line (*P* ≤ 1e-05; −log_10_ *P*-values ≥ 5.00) for *n* = 15,815 U.S. Red Angus beef cattle. A summary of all markers passing the nominal significance threshold is presented in Table 5

To investigate GxE interactions in relation to birth weight, weaning weight, and yearling weight in U.S Red Angus beef cattle, we conducted additional single-marker (836﻿K) analyses. All analyses included a variable for U.S. geographic ecoregion of origin, which was generated via K-means clustering using thirty-year climate data and treated as an interaction term, as previously described [20, 32, 72]. GxE GWAA for birth weight produced evidence for three interactions on BTA26 and BTA22 interacting with two ecoregions (Table 5, Fig. 4; Additional File 1). Positional candidate genes identified by GxE interactions for birth weight have been previously associated with cattle feed efficiency (*PRKG1, LOC531679, SEC61G,* and *NEK10*) (Table S1; Additional File 2) [81–87]. Additionally, *PRKG1* is involved in vasodilation (Table 5) [82]. Notably, only one interaction detected by GxE GWAA for birth weight was identified as a coding variant (Additional File 1). More specifically, the lead SNP within the positional candidate gene *NEK10* encodes a nonsynonymous mutation in exon 2 (Ser → Thr). Four additional interactions were also noted with less statistical support, as described in Table S6 (Additional File 2). Genomic inflation factors for *P*-value estimates obtained from GxE GWAA for birth weight are presented in Table S7 (Additional File 2).

**Table 5:** Summary of GxE interactions detected for birth weight in U.S. Red Angus cattle

Chr_Mb^**a**^	-log_**10**_ P-value	Regression Beta	MAF^**b**^	Supporting SNPs^**c**^	Positional Candidate Genes	Lead SNP Location	Scientific Precedence [reference]; organism; trait
*26_07*^*d*^	7.340	1.350	0.457	9	*PRKG1*	Intron	[49, 62, 81, 82]; Cattle, human, mouse; Conception rate, milk fatty acid, and dry matter intake, vasodilation
*22_01*^*e*^	6.910	−1.082	0.277	9	*LOC531679, SEC61G*	Intergenic	[46, 83–85]; Cattle, human; Milk fatty acid, milk protein percentage, metabolic body weight and feed efficiency, essential for translocation of polypeptides to the ER, role in ER stress response, tumor cell growth
*22_02*^*e*^	5.945	0.951	0.346	5	*NEK10*	Exon^f^	[46, 84, 86, 87]; Human, cattle; Mitotic response to UV-stress, ciliogenesis in development, metabolic body weight, milk protein percentage

^a^ Chromosome_Megabase

^b^ Minor Allele Frequency

^c^ Single Nucleotide Polymorphisms

^d^ Significant for U.S. Arid Prairie Ecoregion

^e^ Significant for U.S. High Plains Ecoregion

^f^ Indicates a predicted nonsynonymous Ser➔Thr, exon 2

**Fig. 5:**
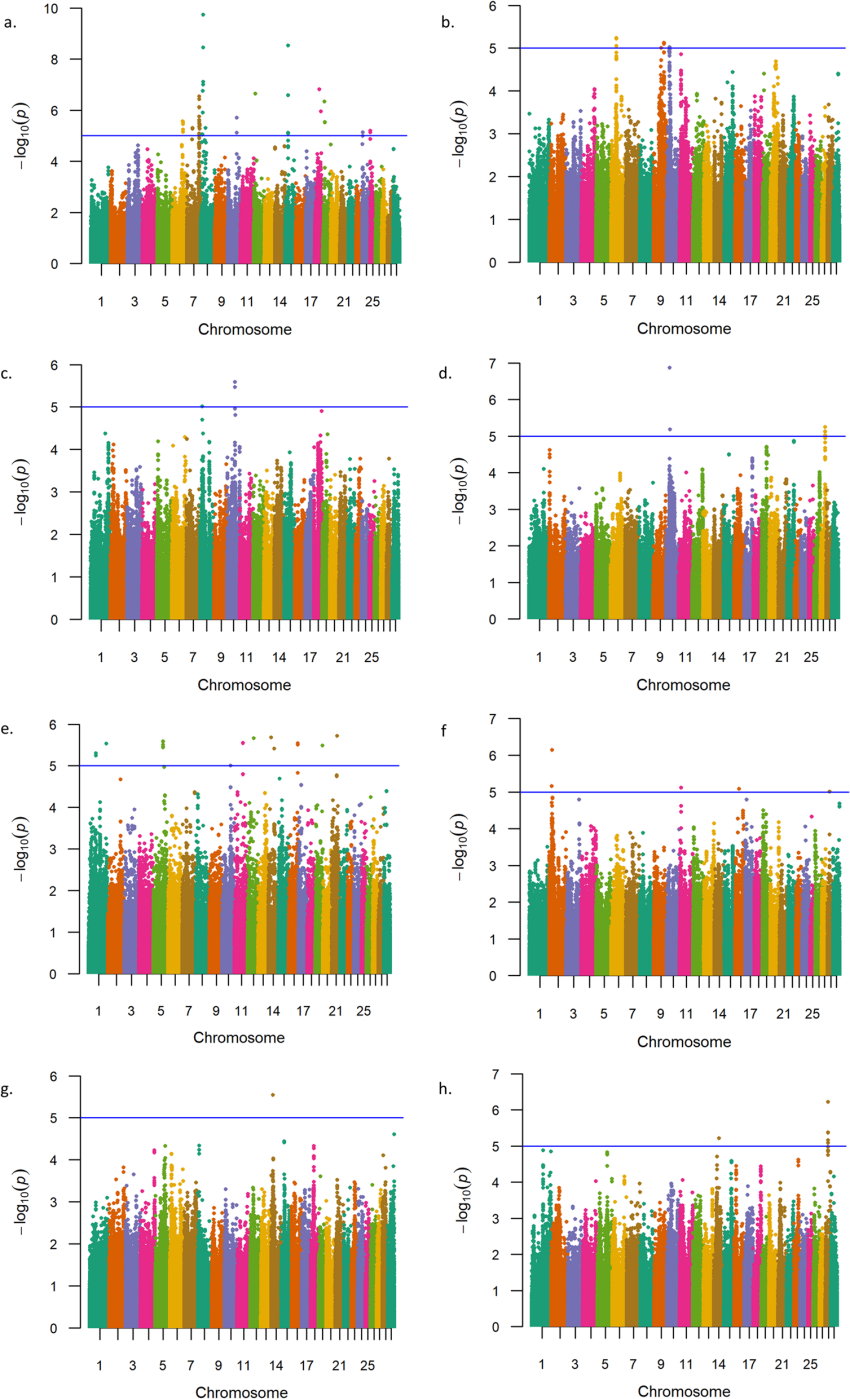
Weaning weight genotype-by-environment interactions. Manhattan plots with -log_10_ *P*-values for U.S. Desert Ecoregion (**a**), U.S. Southeast Ecoregion (**b**), U.S. High Plains Ecoregion (**c**), U.S. Arid Prairie Ecoregion (**d**), U.S. Foothills Ecoregion (**e**), U.S. Forested Mountains Ecoregion (**f**), U.S. Fescue Belt Ecoregion (**g**), and U.S. Upper Midwest and Northeast Ecoregion (**h**). Lead and supporting SNPs for interactions represented at or above the blue line (*P* ≤ 1e-05; −log_10_ *P*-values ≥ 5.00) for *n* = 15,620 U.S. Red Angus beef cattle. A summary of all markers passing the nominal significance threshold is presented in Table 6

GxE GWAA for weaning weight in U.S. Red Angus beef cattle produced evidence for six significant interactions; thereby implicating positional candidate genes related to growth and development (*DNAJC12*), milk production (*LOC112447568, TRNAE-UUC, LOC112447164, COX18*), carcass traits (*LOC112447496, LOC112447497, LOC782092*), cellular proliferation and metabolism (*SIRT1),* and feed efficiency (*LCLAT1*), as defined by relevant lead SNPs (Table 6, Table S1, Fig. 5; Additional File 2) [88–109]. Additionally, positional candidate genes identified on BTA28 (*DNAJC12* and *SIRT1*) have been associated with bovine maturity rate, milk production, and meat quality traits [46, 48, 91]; as well as promoting cellular proliferation and regulation in humans and mice [90, 92]. Interestingly, interactions on BTA7 (101 Mb) and BTA6 (88 Mb) revealed previous associations with aspects of bovine heat stress and thermotolerance, and both showed significant GxE interactions in the U.S. Desert Ecoregion (Table 6; Additional File 1) [89, 104, 105]. All interactions identified in the GxE GWAA for weaning weight in Red Angus cattle were located in noncoding regions (Table 6; Additional File 1). Sixteen additional interactions with less overall statistical support are noted in Table S8, with only one lead SNP encoding a nonsynonymous change within the positional candidate gene *ANKK1* (Table S8; Additional File 1, Additional File 2). Genomic inflation factors for *P*-value estimates obtained from a GxE GWAA for weaning weight are summarized in Table S7 (Additional File 2).

**Table 6:** Summary of GxE interactions detected for weaning weight in U.S. Red Angus cattle

Chr_Mb^**a**^	-log_**10**_ P-value	Regression Beta	MAF^**b**^	Supporting SNPs^**c**^	Positional Candidate Genes	Lead SNP Location	Scientific Precedence [reference]; organism; trait
*7_101*^*d*^	6.537	19.526	0.216	14	*LOC112447568, TRNAE-UUC*	Intergenic	[46, 47, 49, 81, 88, 89]; Cattle, yeast; Milk fatty acid, stature, milk fat yield, and conception rate, protein homeostasis and heat stress adaptation
*28_24*^*e*^	6.213	−6.627	0.404	5	*DNAJC12, SIRT1*	Intergenic	[46–48, 81, 83, 84, 90–93]; Cattle, human; Milk fatty acids, milk fat and protein yield, milk fat and protein percentage, body measurements and meat quality, stature, birth weight, calving ease, maturity rate, cell proliferation, senescence in response to cellular stress
*7_99*^*d*^	5.746	15.175	0.296	5	*LOC112447496, LOC112447497*	Intergenic	[46, 49, 88, 94, 95]; Cattle; Shear force, milk fat yield, and conception rate associations
*11_69*^*f*^	5.550	118.159	0.051	7	*LCLAT1*	Intron	[11, 40, 83, 96–98]; Mouse, cattle; Mitochondrial structure and function, fatty acid oxidation, energy metabolism, obesity and diabetes association, metabolic body weight, milk fatty acid and protein composition, fatty acid associations
*6_88*^*d*^	5.532	42.188	0.014	5	*LOC112447164, COX18*	Intergenic	[46, 49, 84, 99–105]; Cattle, human; Milk, and protein yield, conception rate, mitochondrial biogenesis, oxygen consumption process regulated to adapt to changing environmental conditions
*5_78*^*f*^	5.509	−117.747	0.031	7	*LOC782092*	3’UTR	[62, 106–109]; Cattle; Tenderness association, pregnancy rate, stayability, milk fatty acid

^a^ Chromosome_Megabase

^b^ Minor Allele Frequency

^c^ Single Nucleotide Polymorphisms

^d^ Significant for U.S. Desert Ecoregion

^e^ Significant for U.S. Upper Midwest & Northeast Ecoregion

^f^ Significant for U.S. Foothills Ecoregion

**Fig. 6:**
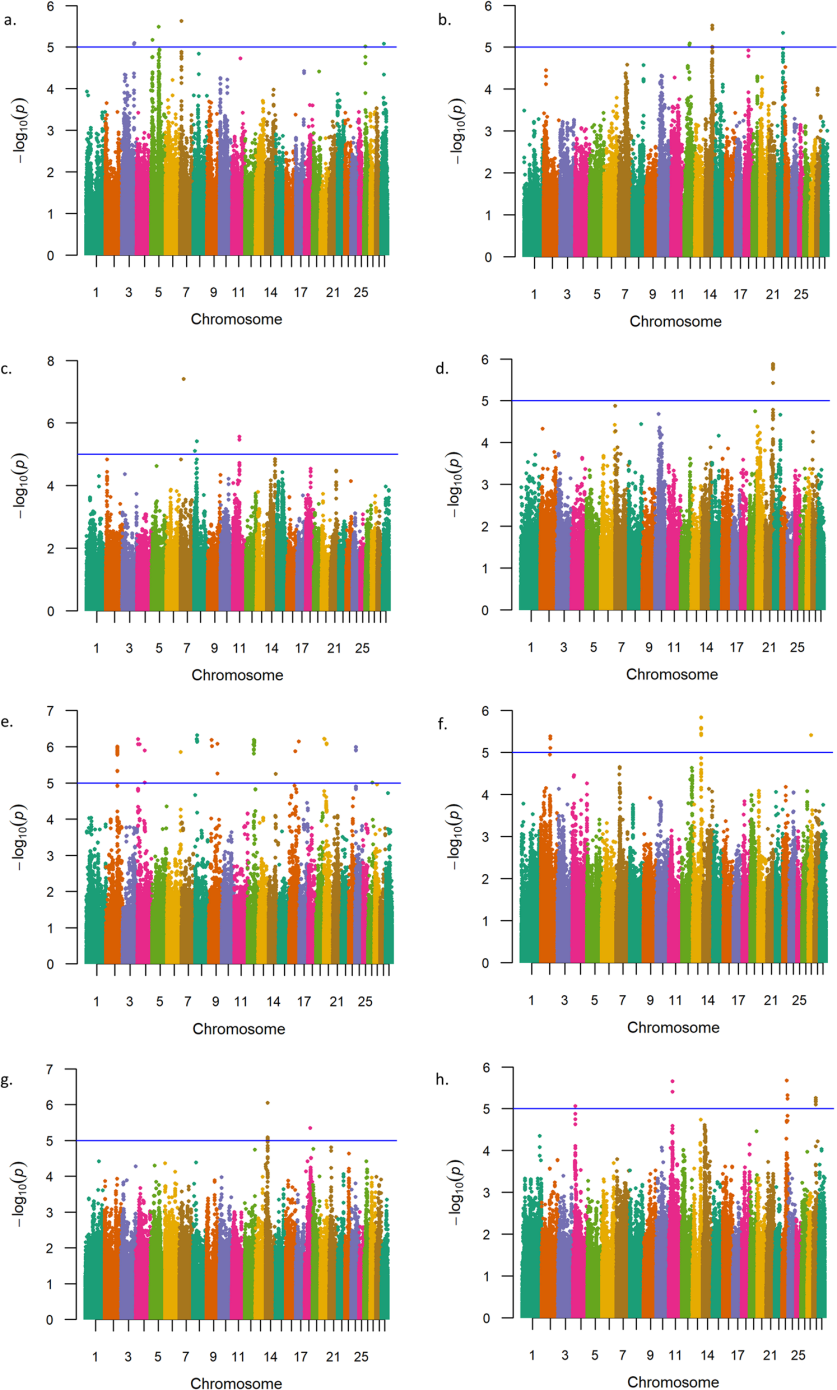
Yearling weight genotype-by-environment interactions. Manhattan plots with -log_10_ *P*- values for U.S. Desert Ecoregion (**a**), U.S. Southeast Ecoregion (**b**), U.S. High Plains Ecoregion (**c**), U.S. Arid Prairie Ecoregion (**d**), U.S. Foothills Ecoregion (**e**), U.S. Forested Mountains Ecoregion (**f**), U.S. Fescue Belt Ecoregion (**g**), and U.S. Upper Midwest and Northeast Ecoregion (**h**). Lead and supporting SNPs for interactions represented at or above the blue line (*P* ≤ 1e-05; −log_10_ *P*-values ≥ 5.00) for *n* = 12,388 U.S. Red Angus beef cattle. A summary of all markers passing the nominal significance threshold is presented in Table 7

We conducted a final GxE GWAA for yearling weight in U.S. Red Angus beef cattle and found evidence for five interactions which met a nominal significance threshold for polygenic traits (*P ≤* 1e-05), as summarized in Table 7 and Fig. 6 (Additional File 1) [71]. The positional candidate genes corresponding to each interaction have been associated with cellular proliferation and differentiation (*EDNRB, POU4F1,* and *DNAJC12*), bovine development (*PARD3B, NRP2,* and *SIRT1*), neural development (*PARD3B* and *NRP2*), and carcass traits (*ZHX3*) (Table 7, Table S1; Additional File 2) [110–125]. All lead SNPs defining GxE associations for yearling weight via GWAA were located in noncoding regions. Positional candidate genes underlying an interaction detected on BTA28 at 24 Mb (i.e., *DNAJC1* and *SIRT1*) strongly suggest that pleiotropic GxE interactions exist with respect to weaning weight (Table 6) and yearling weight (Table 7) for U.S. Red Angus beef cattle. Moreover, pleiotropic GxE interactions for weaning weight and yearling weight were also detected on BTA8 at 15 Mb, and on BTA27 at 39 Mb, including positional candidate genes *LINGO2*, *LOC112447774*, and *PSD3* (Tables S8-S9; Additional File 2), but with less overall statistical support. All pleiotropic associations detected for U.S. Red Angus beef cattle are summarized in Table S1 (Additional File 2). Genomic inflation factors for *P-*value estimates obtained from a GxE GWAA for yearling weight are displayed in Table S7 (Additional File 2).

**Table 7:** Summary of GxE interactions detected for yearling weight in U.S. Red Angus cattle

Chr_Mb^**a**^	-log_**10**_ P-value	Regression Beta	MAF^**b**^	Supporting SNPs^**c**^	Positional Candidate Genes	Lead SNP Location	Scientific Precedence [reference]; organism; trait
*12_54*^*d*^	6.173	207.987	0.027	5	*EDNRB, POU4F1*	Intergenic	[24, 40, 49, 81, 101, 110–115]; Mouse, cattle, human; Neural crest cell proliferation, milk fatty acid, residual feed intake, dry matter intake, conception rate, weaning weight, vasodilation, neural cell fate specification, cardiac development
*2_93*^*d*^	6.002	−204.602	0.021	46	*PARD3B*	Intron	[40, 116–118]; Mouse, cattle; Neurogenesis and cortical development, cell proliferation and differentiation, dendritic spine formation, average daily gain
*2_94*^*d*^	5.957	−203.686	0.034	15	*NRP2*	Intron	[40, 46, 119–121]; Mouse, cattle; Neural development and formation of the central and peripheral nervous systems, vascular development, average daily gain, milk fat yield
*13_70*^*e*^	5.833	11.315	0.295	6	*ZHX3*	Intron	[88, 94, 122–125]; Rat, human, cattle; Development of nephritic syndrome, regulation of metabolism, weaning weight, shear force, tenderness response to stress, scrotal circumference, herd life, rump fat thickness, conception rate
*28_24*^*f*^	5.258	−9.747	0.470	7	*DNAJC12, SIRT1*	Intergenic	[46–48, 81, 83, 84, 90–93]; Cattle, human; Milk fatty acids, milk fat and protein yield, milk fat and protein percentage, body measurements and meat quality, stature, birth weight, calving ease, maturity rate, cell proliferation, senescence in response to cellular stress

^a^ Chromosome_Megabase

^b^ Minor Allele Frequency

^c^ Single Nucleotide Polymorphisms

^d^ Significant for U.S. Foothills Ecoregion

^e^ Significant for U.S. Forested Mountains Ecoregion

^f^ Significant for U.S. Upper Midwest & Northeast Ecoregion

## Conclusions

Herein, we present evidence for pleiotropic QTL resulting from traditional GWAA influencing birth weight, weaning weight, and yearling weight in U.S. Red Angus beef cattle, and further confirm the involvement of genomic regions on BTA6 from 34 to 41 Mb and BTA14 from 23 to 25 Mb in various aspects of bovine growth, feed efficiency, carcass traits, and stature across breeds. Additionally, the results from our GWAA and GxE GWAA for birth weight, weaning weight, and yearling weight in U.S. Red Angus beef cattle provide compelling comparative evidence for at least 35 statistically well-supported associations segregating across multiple cattle breeds; including three U.S. Red Angus growth associations that were previously detected for mid-test metabolic weight, two QTL previously detected for average daily gain in U.S. SimAngus and Hereford beef cattle, and 11 U.S. Red Angus growth associations also detected in U.S. Gelbvieh beef cattle. Examination of GxE GWAA associations for U.S. Red Angus growth traits revealed ecoregion-specific positional candidate genes with suggested pleiotropy for genes *DNAJC12,* and *SIRT1* for weaning weight and yearling weight. Similar to previous GWAA and GxE GWAA on U.S. Gelbvieh and Simmental beef cattle, significant GxE associations detected for birth weight, weaning weight, and yearling weight in U.S. Red Angus cattle were not overlapping; thereby suggesting that although the majority of the main effect QTL were conserved between breeds, GxE interactions were not conserved. In agreement with previous GxE and local adaptation results in beef cattle, we find GxE effects associated with vasodilation and neural development. Identification of pleiotropic growth QTL and breed specific GxE interactions may potentially serve to benefit beef breeding programs across diverse U.S. climates via creation of region-specific genomic predictions. Moreover, the results of this study further demonstrate that imputation to a union set of high-density SNPs (i.e., 836K) can directly facilitate future studies at a fraction of the cost associated with direct genotyping; thus providing a research framework that directly enables large-scale analyses for economically important livestock species, and the potential for identifying causal variants via genome sequence-level imputed genotypes.

## Methods

Birth weight (*n* = 17,320; in 5803 contemporary groups), weaning weight (*n* = 17,306; in 6478 contemporary groups), and yearling weight (*n* = 13,648; in 4809 contemporary groups) phenotypes were obtained from the Red Angus Association of America for animals with genotypes. These animals had 2102 unique sires and 12,124 unique dams. Analyzed animals were born from 1975 to 2017. Phenotypes were pre-adjusted by Red Angus Association of America for age of animal and age of the dam (i.e., 205-day weight for weaning weight). Phenotypes were further adjusted using the mmer() function in the sommer package v3.9.3 in R v3.5.2 [126, 127] to account for contemporary group effects using contemporary group IDs supplied by Red Angus Association of America. Details regarding Red Angus contemporary groups are summarized in Additional File 1. Birth weight and weaning weight were adjusted for maternal effect using pedigree files provided by the Red Angus Association of America. Discrete climate ecoregions were designated for each individual using K-means clustering with three continuous climate variables (mean temperature in Fahrenheit, precipitation in inches, and elevation in feet) gathered from the PRISM climate dataset from thirty years of normalized records [128]. The pamk function in conjunction with the kmeans algorithm in the fpc (Flexible Procedures for Clustering) [129] package and the RStoolbox package [127, 130] in R assigned every four kilometer (km) square of the continental U.S. to one of 9 clusters, denoted as ecoregions. These designated ecoregions consist of the Upper Midwest & Northeast, Fescue Belt, Rainforest, Forested Mountains, High Plains, Foothills, Desert, Southeast, and Arid Prairie. Animals were assigned to ecoregions by breeder zip-code as recorded in the U.S. Red Angus Association of America herdbook [32]. If the breeder’s zip-code overlapped with two or more ecoregions, the animal was filtered from further analysis.

Genotypes from 22,932 U.S. Red Angus cattle were provided by Neogen GeneSeek (Lincoln, NE, U.S.A). The ARS-UCD1.2 *Bos taurus* assembly [131] was used for SNP positions. The genotypes underwent filtering using PLINK 1.9 to remove individuals with call rates < 0.90 on an assay-by-assay basis (i.e., GeneSeek GGP-LDv3, GeneSeek GGP-LDv4, GeneSeek GGP-90KT, GeneSeek GGP-HDv3, GeneSeek Bovine-GGP-F250, Illumina Bovine SNP50, and Illumina HD 778K), removal of variants with call rates < 0.90 and Hardy-Weinberg Equilibrium (HWE) *P*-values < 1e-20 to exclude poorly genotyped loci [132]. Only autosomal chromosomes were utilized in these analyses. The remaining 22,457 individuals and associated genotypes were then merged and phased using PLINK and EagleV2.4, respectively [133]. Phased haplotypes for 8622 diverse individuals genotyped using the Illumina HD (778K SNPs; Illumina, San Diego, CA) and 28,114 individuals genotyped using the Bovine-GGP-F250 (250K SNPs; GeneSeek, Lincoln, NE) were used as a multi-breed reference panel for imputation in minimac4 as previously described [19, 134]. The 22,457 Red Angus genotypes from various assays were imputed for all markers contained on the two high-density research chips in this multi-breed reference panel. A total of 6642 cattle had only genotype information, thus providing 15,815 individuals with 836,118 markers each (ARS-UCD1.2) to be utilized as the final dataset for GWAA and GxE GWAA. Minimac4 reported imputed dosage genotypes to account for any potential uncertainty during imputation processes, as previously described [19, 134].

Imputed genotypes (836K markers) and the adjusted phenotypes for Red Angus cattle were used to conduct univariate linear mixed model GWAA for birth weight (15,815 individuals), weaning weight (15,620 individuals), and yearling weight (12,388 individuals) using the program GEMMA. Prior to the execution of all GWAA, GEMMA filtered all SNP loci as follows: MAF (< 0.01 excluded), polymorphism (monomorphic SNPs excluded), and Hardy-Weinberg Equilibrium (HWE; *P*-values < 0.001 excluded), thereby producing genotypic sets of 675,115 SNPs for birth weight, 675,060 SNPs for weaning weight, and 674,493 SNPs for yearling weight. Genomic relationship matrices (*G*_*s*_) were computed with the imputed genotypes in GEMMA to control for dependence between samples due to relatedness. The linear mixed models implemented in GEMMA also estimate the proportion of variance explained (PVE) by the genomic relationship matrix. The PVE is also referred to as “chip heritability” [135]. The univariate linear mixed model implemented for GWAA can be generally specified as: *y* = *Wα* + *xβ* + *u* + *ϵ*; where *y* is a *n*-vector of quantitative traits (i.e., birth weight, weaning weight, and yearling weight) for *n*-Red Angus individuals, *W* is an *n* x *c* matrix of specified covariates (i.e., fixed effects) including a column of 1﻿s, *α* is a *c*-vector of corresponding coefficients including the intercept, *x* is an *n*-vector of SNP genotypes, *β* is the effect size of the SNP, *u* is an *n*-vector of random effects, and *ϵ* represents an *n*-vector of errors [20, 135]. Additionally, *u* ∼ *MVN*_*n*_(0, *λτ*^−1^Κ) and *ϵ* ∼ *MVN*_*n*_(0, *τ*^−1^*I*), where *MVN* denotes multivariate normal distribution, *τ*^−1^ is the variance of the residual errors, *λ* is the ratio between the two variance components, Κ is the *n* x *n* genomic relatedness matrix, and *I* represents an *n* x *n* identity matrix [20, 135]. Specifically, GEMMA performed a Wald test using -lmm 1 as follows: ${F}_{Wald}=\frac{{\hat{\beta}}^2}{V\left(\hat{\beta}\right)}$, tests the alternative hypothesis (${H}_1:\hat{\beta}\ne 0\Big)$ for each SNP against the null hypothesis for each SNP (${H}_0:\hat{\beta}=0$). Moreover, $\hat{\beta}={\left({x}^T{P}_c\left({\hat{\lambda}}_r\right)x\right)}^{-1}\left({x}^T{P}_c\left({\hat{\lambda}}_r\right)y\right)$ is the estimate for *β* obtained using the restricted maximum likelihood (REML) estimate ${\hat{\lambda}}_r$ in the alternative model; and $V\ \left(\hat{\beta}\right)={\left(n-c-1\right)}^{-1}{\left({x}^T{P}_c\left({\hat{\lambda}}_r\right)x\right)}^{-1}\left({y}^T{P}_x\left({\hat{\lambda}}_r\right)y\right)$ is the variance for $\hat{\beta}$ [20, 135]. Under the null hypothesis, the Wald test statistics (*F*_*Wald*_) come from an *F*(1, *n* − *c* − 1) distribution [20, 135]; with GEMMA producing marker-based REML estimates and corresponding *P*-values. For all GxE GWAAs, discrete geographic ecoregion (i.e., the environmental variable) was specified as an interaction term using the -gxe command. GxE GWAA were computed using a mixed model which can be generally specified as: *y* = *Wα* + *x*_*snp*_*β*_*snp*_ + *x*_*env*_*β*_*env*_ + *x*_*snp*_ × *x*_*env*_*β*_*snp* × *env*_ + *u* + *ϵ*; where *y* is an *n*-vector of quantitative traits (i.e., birth weight, weaning weight, and yearling weight) for *n*-Red Angus individuals, *W* represents an *n* x *c* matrix of specified covariates, *α* is a *c*-vector of corresponding coefficients including the intercept, *x*_*snp*_ represents an *n*-vector of SNP genotypes, *β*_*snp*_ is the effect size of the SNP, *x*_*env*_ is an *n*-vector of membership in a single ecoregion, *β*_*env*_ represents the fixed effect of the ecoregion, *β*_*snp* × *env*_ is the estimated interaction between SNP genotype and ecoregion, *u* is an *n*-vector of random effects, and *ϵ* is an *n*-vector of errors [20, 135]. As above, *u* ∼ *MVN*_*n*_(0, *λτ*^−1^Κ) and *ϵ* ∼ *MVN*_*n*_(0, *τ*^−1^*I*). Each discrete ecoregion was compared against the remaining U.S. dataset using binary (0, 1) coding as an environmental variable with one exception; the Rainforest ecoregion had insufficient sample size for GxE GWAA, and thus eight separate GxE GWAA were computed (Additional File 1). GEMMA evaluated the alternative hypothesis for each interaction (*H*_1_ : *β*_*snp* × *env*_ ≠ 0) in comparison to the null hypothesis (*H*_0_ : *β*_*snp* × *env*_ = 0) using linear mixed models while controlling for population stratification, SNP main effect, and environmental effect while examining the interaction effect of each ecoregion [20, 135]. Single-marker *P*-value results produced by GEMMA using the -lmm 1 and -gxe commands were further adjusted using chi-squared test statistics divided by a constant for additional genomic control [37, 136]. Adjusted *P-*value results were utilized to produce Manhattan plots using the manhattan command in R [137]. All SNPs meeting the nominal significance threshold (*P* ≤ 1e-05) were rounded to the nearest Mb and strongly supported QTL were defined by ≥ 5 significant SNP loci with MAF ≥ 0.01 (i.e., a lead SNP plus four or more additional supporting SNPs within the same rounded Mb) [31, 71]. Additional QTL were also noted with less overall statistical support in Tables S2, S4-S6, and S8-S9; thereby representing QTL defined by ≥ 2 but ≤ 4 SNP loci which met the nominal significance threshold (*P* ≤ 1e-05) within the same rounded Mb (Additional File 2). Positional candidate genes were implicated by location of the lead SNP. Genomic inflation factors (λ) were estimated using observed and expected *P*-values via regression for all GWAA and GxE GWAA in R [80, 138, 139]. The proportion of variance explained (PVE) by bovine SNPs was estimated as previously described [140]. Genetic correlations were estimated using the multivariate approach implemented in GEMMA [135, 141], as previously described [142].

## Supplementary Information

**Additional file 1:** Description and acronym definitions for summary data. Summary data for all genome-wide association analyses (GWAA) and genotype-by-environment genome-wide association analyses (GxE GWAA) performed for bovine birth weight, weaning weight, and yearling weight in tabular format.

**Additional file 2: Table S1.** Summary of QTL supporting pleiotropy detected for birth weight, weaning weight, and yearling weight GWAA and GxE GWAA in U.S. Red Angus cattle. **Table S2.** Summary of QTL with 2 to 4 supporting SNPs detected for birth weight in U.S. Red Angus cattle. **Table S3.** Genomic inflation factors (λ) calculated using observed *P*-values and expected *P*-values for GWAA for growth traits in U.S. Red Angus beef cattle. **Table S4.** Summary of QTL with 2 to 4 supporting SNPs detected for weaning weight in U.S. Red Angus cattle. **Table S5.** Summary of QTL with 2 to 4 supporting SNPs detected for yearling weight in U.S. Red Angus cattle. **Table S6.** Summary of GxE interactions with 2 to 4 supporting SNPs detected for birth weight in U.S. Red Angus cattle. **Table S7.** Genomic inflation factors (λ) calculated using observed *P*-values and expected *P*-values for GxE GWAA for growth traits in U.S. Red Angus beef cattle. **Table S8.** Summary of GxE interactions with 2 to 4 supporting SNPs detected for weaning weight in U.S. Red Angus cattle. **Table S9.** Summary of GxE interactions with 2 to 4 supporting SNPs detected for yearling weight in U.S. Red Angus cattle.

## Data Availability

Third party data analyzed in the present study are available for non-commercial use via data use agreement (DUA) with the Red Angus Association of America.
