# Supplementary material for: Genome-wide association and genotype by environment interactions for growth traits in U.S. Red Angus cattle
Source: BMC Genomics. 2022 Jul 16;23:517. doi: 10.1186/s12864-022-08667-6 (PMC9287884; doi:10.1186/s12864-022-08667-6)
Supplement: Supplementary file 2 — Additional file 2: Table S1. Summary of QTL supporting pleiotropy detected for birth weight, weaning weight, and yearling weight GWAA and GxE GWAA in U.S. Red Angus cattle. Table S2. Summary of QTL with 2 to 4 supporting SNPs detected for birth weight in U.S. Red Angus cattle. Table S3. Genomic inflation factors (λ) calculated using observed P-values and expected P-values for GWAA for growth traits in U.S. Red Angus beef cattle. Table S4. Summary of QTL with 2 to 4 supporting SNPs detected for weaning weight in U.S. Red Angus cattle. Table S5. Summary of QTL with 2 to 4 supporting SNPs detected for yearling weight in U.S. Red Angus cattle. Table S6. Summary of GxE interactions with 2 to 4 supporting SNPs detected for birth weight in U.S. Red Angus cattle. Table S7. Genomic inflation factors (λ) calculated using observed P-values and expected P-values for GxE GWAA for growth traits in U.S. Red Angus beef cattle. Table S8. Summary of GxE interactions with 2 to 4 supporting SNPs detected for weaning weight in U.S. Red Angus cattle. Table S9. Summary of GxE interactions with 2 to 4 supporting SNPs detected for yearling weight in U.S. Red Angus cattle. [file 12864_2022_8667_MOESM2_ESM.docx]

**Supplemental Information**

**Table S1.** Summary of QTL with five or more supporting SNPs detected for birth weight, weaning weight, and yearling weight GWAA and GxE GWAA in U.S. Red Angus cattle compared to previous studies.

| **All QTL**  **(Chr_Mb)** | **Within-study evidence for pleiotropy influencing Red Angus growth traits** | **Evidence for pleiotropy influencing Gelbvieh growth traits ^a^** | **Evidence for pleiotropy influencing bovine growth, feed efficiency, production, and/or carcass traits ^b^** |
| --- | --- | --- | --- |
| 2_93 |  |  | X |
| 2_94 |  |  | X |
| 5_78 |  |  | X |
| 6_32 | X |  | X |
| 6_33 |  |  | X |
| 6_34 | X | X | X |
| 6_35 | X |  | X |
| 6_36 | X | X | X |
| 6_37 | X | X | X |
| 6_38 | X | X | X |
| 6_39 | X | X | X |
| 6_40 | X | X | X |
| 6_41 | X | X | X |
| 6_88 |  |  | X |
| 7_90 |  |  | X |
| 7_91 | X |  | X |
| 7_99 |  |  | X |
| 7_101 |  |  | X |
| 11_69 |  |  | X |
| 12_54 |  |  | X |
| 13_70 |  |  | X |
| 14_02 |  |  | X |
| 14_22 |  |  | X |
| 14_23 | X | X | X |
| 14_24 | X | X | X |
| 14_25 |  | X | X |
| 20_03 |  |  |  |
| 20_04 | X |  | X |
| 20_05 | X | X | X |
| 20_06 | X |  | X |
| 20_07 |  |  | X |
| 22_01 |  |  | X |
| 22_02 |  |  | X |
| 26_07 |  |  | X |
| 28_24 | X |  | X |
| **Totals:** | **16** | **11** | **34** |

^a^ Obtained from Smith et al. 2019 [23]

^b^ Refer to Tables 2-7

**Table S2.** Summary of QTL with 2 to 4 supporting SNPs detected for birth weight in U.S. Red Angus cattle.

| **Chr_Mb** | **-log_10_**  **P-value** | **Regression**  **Beta** | **MAF** | **Supporting**  **SNPs** | **Positional**  **Candidate**  **Genes** | **Lead SNP**  **Location** | **Scientific Precedence [reference]; organism; trait** |
| --- | --- | --- | --- | --- | --- | --- | --- |
| *6_42* | 9.560 | -1.621 | 0.056 | 3 | *LOC782172, ADGRA3* | Intergenic | [1–5]; Cattle; Birth, weaning, and yearling weight, calving ease, yield grade, mature weight, conception rate, body condition score |
| *20_03* | 7.565 | -0.813 | 0.402 | 4 | *FGF18, LOC112443066* | Intergenic | [6]; Cattle; Cell cycle regulation of granulosa cells |
| *7_18* | 5.869 | 0.958 | 0.183 | 3 | *SH2D3A* | Intron | [7]; Cattle; Milk yield |

**Table S3.** Genomic inflation factors (λ) calculated using observed *P-*values and expected *P-*values for GWAA for growth traits in U.S. Red Angus beef cattle.

| **Trait** | **λ** | **SE (λ)** |
| --- | --- | --- |
| **Birth Weight** | 1.299 | 0.002 |
| **Weaning Weight** | 1.112 | 0.001 |
| **Yearling Weight** | 1.222 | 0.001 |

**Table S4.** Summary of QTL with 2 to 4 supporting SNPs detected for weaning weight in U.S. Red Angus cattle.

| **Chr_Mb** | **-log_10_**  **P-value** | **Regression**  **Beta** | **MAF** | **Supporting**  **SNPs** | **Positional**  **Candidate**  **Genes** | **Lead SNP**  **Location** | **Scientific Precedence [reference]; organism; trait** |
| --- | --- | --- | --- | --- | --- | --- | --- |
| *6_42* | 6.421 | -7.430 | 0.056 | 4 | *LOC782172, ADGRA3* | Intergenic | [1–5]; Cattle; Birth, weaning, and yearling weight, calving ease, yield grade, mature weight, conception rate, body condition score |
| *14_35* | 6.377 | -4.070 | 0.266 | 2 | *LOC783431, LOC112449626,*  *EYA1* | Intergenic | [8, 9]; Cattle; Transcription factor expressed in hypothalamus, controls energy balance relating to puberty |
| *6_33* | 5.996 | -4.061 | 0.299 | 4 | *CCSER1* | Intron | [1, 3, 10]; Cattle, human; Carcass weight, fat thickness, conception rate, regulator of mitosis |
| *14_33* | 5.665 | 3.932 | 0.279 | 2 | *SLCO5A1* | Exon^a^ | [11, 12]; Frog, pig; Expressed in mature dendritic cells, maintenance of pregnancy |
| *14_34* | 5.449 | 3.402 | 0.460 | 4 | *LACTB2* | Intron | [13, 14]; Human, cattle; Essential to mitochondrial function, milk yield |
| *7_91* | 5.443 | 3.908 | 0.219 | 3 | *LOC112447489* | Intron | [7]; Cattle; Milk protein yield |

^a^ Indicates a predicted synonymous mutation Arg → Arg, exon 6

**Table S5.** Summary of QTL with 2 to 4 supporting SNPs detected for yearling weight in U.S. Red Angus cattle.

| **Chr_Mb** | **-log_10_**  **P-value** | **Regression**  **Beta** | **MAF** | **Supporting**  **SNPs** | **Positional**  **Candidate**  **Genes** | **Lead SNP**  **Location** | **Scientific Precedence [reference]; organism; trait** |
| --- | --- | --- | --- | --- | --- | --- | --- |
| *6_42* | 8.618 | -15.364 | 0.056 | 3 | *LOC782172, ADGRA3* | Intergenic | [1–5]; Cattle; Birth, weaning, and yearling weight, calving ease, yield grade, mature weight, conception rate, body condition score |
| *20_02* | 5.526 | -6.398 | 0.400 | 2 | *DOCK2* | Intron | [15, 16]; Mouse; Roles in migration and activation of T cells |
| *21_66* | 5.295 | 4.889 | 0.426 | 4 | *MEG9, LOC112443172* | Intergenic | [17, 18]; Human, cattle; Cell proliferation and viability, natural antibodies in blood |
| *7_90* | 5.167 | 8.377 | 0.107 | 2 | *ADGRV1* | Intron | [3, 19, 20]; Cattle, mouse; Birth and yearling weights, conception rate, regulation of bone mineral density and fragility |
| *6_32* | 5.094 | 7.595 | 0.269 | 3 | *GRID2* | Intron | [7, 19, 21, 22]; Human, cattle; Mammalian nervous system mediation, dry matter intake, average daily gain, birth weight, milk fat yield |

**Table S6.** Summary of GxE interactions with 2 to 4 supporting SNPs detected for birth weight in U.S. Red Angus cattle.

| **Chr_Mb** | **-log_10_**  **P-value** | **Regression**  **Beta** | **MAF** | **Supporting**  **SNPs** | **Positional**  **Candidate**  **Genes** | **Lead SNP**  **Location** | **Scientific Precedence [reference]; organism; trait** |
| --- | --- | --- | --- | --- | --- | --- | --- |
| *12_33^a^* | 5.717 | 3.409 | 0.068 | 2 | *LOC536660* | Intron | [1, 7, 23]; Cattle; Age of puberty, protein yield, carcass weight, calf survival |
| *22_10^b^* | 5.610 | 2.694 | 0.089 | 4 | *MIR128-2, LOC104975489* | Intergenic | [7, 24, 25]; Cattle, mouse; Conception rate, milk and protein yields, body temperature under climatic stress, brain and nervous system development and maintenance of function, brain aging and neurodegeneration processes |
| *2_45^c^* | 5.258 | -2.305 | 0.290 | 4 | *RBM43, LOC107132261* | Intergenic | [26, 27]; Human, cattle; Developmentally regulates mRNA processing, association with type II diabetes, milk, fat, and protein yields |
| *2_12^a^* | 5.241 | 1.547 | 0.459 | 3 | *ZNF804A* | Intron | [28–31]; Human, cattle; Role in signaling pathways for neuron differentiation, cell adhesion, muscular traits, expression and splicing master regulators for meat quality, fatty acid, back length |

^a^ Significant for U.S. Southeast Ecoregion

^b^ Significant for U.S. Arid Prairie Ecoregion

^c^ Significant for U.S. Desert Ecoregion

**Table S7.** Genomic inflation factors (λ) calculated using observed *P-*values and expected *P-*values for GxE GWAA for growth traits in U.S. Red Angus beef cattle.

| **Trait** | **λ** | **SE (λ)** |
| --- | --- | --- |
| **BW U.S Desert Ecoregion** | 0.993 | 9.013E-06 |
| **BW U.S. Southeast Ecoregion** | 1.007 | 9.188E-06 |
| **BW U.S. High Plains Ecoregion** | 1.019 | 1.815E-05 |
| **BW U.S. Arid Prairie Ecoregion** | 1.002 | 2.081E-05 |
| **BW U.S. Foothills Ecoregion** | 1.026 | 2.262E-05 |
| **BW U.S. Forested Mountains Ecoregion** | 0.998 | 1.299E-05 |
| **BW U.S. Fescue Belt Ecoregion** | 0.996 | 1.717E-05 |
| **BW U.S. Upper Midwest and Northeast Ecoregion** | 0.981 | 1.395E-05 |
| **WW U.S. Desert Ecoregion** | 1.014 | 4.974E-05 |
| **WW U.S. Southeast Ecoregion** | 1.010 | 1.989E-05 |
| **WW U.S. High Plains Ecoregion** | 0.999 | 1.079E-05 |
| **WW U.S. Arid Prairie Ecoregion** | 0.982 | 1.193E-05 |
| **WW U.S. Foothills Ecoregion** | 1.045 | 2.905E-05 |
| **WW U.S. Forested Mountains Ecoregion** | 1.016 | 1.194E-05 |
| **WW U.S. Fescue Belt Ecoregion** | 0.986 | 2.164E-05 |
| **WW U.S. Upper Midwest and Northeast Ecoregion** | 1.008 | 1.008E-05 |
| **YW U.S. Desert Ecoregion** | 1.021 | 1.966E-05 |
| **YW U.S. Southeast Ecoregion** | 1.019 | 2.792E-05 |
| **YW U.S. High Plains Ecoregion** | 1.013 | 1.985E-05 |
| **YW U.S. Arid Prairie Ecoregion** | 0.995 | 1.377E-05 |
| **YW U.S. Foothills Ecoregion** | 1.016 | 7.849E-05 |
| **YW U.S. Forested Mountains Ecoregion** | 1.003 | 1.128E-05 |
| **YW U.S. Fescue Belt Ecoregion** | 1.022 | 1.145E-05 |
| **YW U.S. Upper Midwest and Northeast Ecoregion** | 1.022 | 1.316E-05 |

**Table S8.** Summary of GxE interactions with 2 to 4 supporting SNPs detected for weaning weight in U.S. Red Angus cattle.

| **Chr_Mb** | **-log_10_**  **P-value** | **Regression**  **Beta** | **MAF** | **Supporting**  **SNPs** | **Positional**  **Candidate**  **Genes** | **Lead SNP**  **Location** | **Scientific Precedence [reference]; organism; trait** |
| --- | --- | --- | --- | --- | --- | --- | --- |
| *8_20^a^* | 8.443 | -91.905 | 0.013 | 3 | *LOC616508, ELAVL2* | Intergenic | [3, 7, 32–34]; Cattle, human; Early development, conception rate, milk fatty acids, milk fat and protein yield, daughter stillbirth, neuronal development and function, translational repressor during oocyte growth |
| *15_24^a^* | 6.588 | -74.087 | 0.012 | 2 | *ANKK1* | Exon^b^ | [3, 7, 35, 36]; Human, mouse, cattle; Neurogenesis, insulin sensitivity and obesity risk, embryonic myogenesis and muscle regeneration, milk yield, conception rate |
| *10_73^a^* | 5.707 | -36.843 | 0.049 | 2 | *LOC112448663, LOC515823* | Intergenic | [1, 37–40]; Cattle; Stature, calving ease, rump angle, ketosis, milk fat yield, conception rate |
| *5_60^c^* | 5.592 | 118.728 | 0.024 | 3 | *TMTC1* | Intron | [41, 42]; Human; Controlling cellular metabolism, proliferation, and differentiation, regulation of glucose homeostasis |
| *10_63^d^* | 5.584 | -6.643 | 0.235 | 3 | *LOC112448561, LOC782100* | Intergenic | [3, 38, 43]; Cattle; Conception rate, rump angle |
| *16_75^c^* | 5.543 | 19.687 | 0.305 | 2 | *LOC112441795, LOC112441796* | Intergenic | [1, 3, 7, 14]; Cattle; Somatic cell score, milk protein yield, conception rate, ribeye area |
| *19_15^a^* | 5.526 | -60.631 | 0.021 | 2 | *UNC45B* | Intron | [7, 40, 44]; Human, cattle; Striated muscle formation, eye development, milk fat yield, conception rate |
| *7_98^a^* | 5.474 | 17.984 | 0.161 | 3 | *LOC104969002, LOC100847536* | Intergenic | [40, 45–50]; Cattle; Shear force, temperature associated thermotolerance, conception rate, intramuscular fat percentage, residual feed intake |
| *1_61^c^* | 5.304 | -95.209 | 0.013 | 2 | *LSAMP, TRNAC-GCA* | Intergenic | [3, 31, 51–54]; Rats, mouse, cattle; Formation of neuronal connections during development of the limbic system, conception rate, milk fatty acid, methane production and digestion, dry matter intake, residual feed intake |
| *7_44^a^* | 5.301 | -35.491 | 0.027 | 3 | *TCF3, LOC781086* | Intergenic | [3, 7, 55–57]; Mouse, zebrafish, cattle, human; Embryonic development, cell maintenance and differentiation in the central nervous system, regulator of muscle tissue, puberty onset, milk fat yield, conception rate, osteogenesis |
| *27_39^e^* | 5.248 | 8.056 | 0.436 | 4 | *PSD3* | Intron | [2, 3, 7, 33, 38, 58–60]; Human, cattle; Obesity and type II diabetes, milk fat percentage, mature weight, body height, intramuscular fat, shear force, conception rate |
| *6_42^f^* | 5.236 | 9.807 | 0.443 | 3 | *ADGRA3* | Intron | [1–4, 19, 33, 43, 54]; Cattle; Milk fatty acid, yield grade, birth weight, calving ease, conception rate, body condition score, milk yield, mature weight |
| *14_44^g^* | 5.217 | 19.114 | 0.024 | 2 | *LOC101905345, LOC112449520* | Intergenic | [14, 39, 43, 61, 62]; Cattle; Conception rate, stillbirth, scrotal circumference, somatic cell score, residual feed intake |
| *25_02^a^* | 5.194 | -59.160 | 0.023 | 2 | *SYNGR3* | 3'UTR | [7, 45, 48, 63]; Mouse, cattle; Conception rate, calf survival, milk and fat yield, age at puberty, shear force |
| *9_84^f^* | 5.121 | 23.636 | 0.054 | 2 | *GRM1* | Intron | [31, 64]; Cattle; Fatty acid in muscle, milk energy |
| *8_15^a^* | 5.049 | 71.649 | 0.013 | 2 | *LINGO2* | 5'UTR | [2, 59]; Cattle; Milk fatty acids, mature weight |

^a^ Significant for U.S. Desert Ecoregion,

^b^ Indicates a nonsynonymous mutation Arg → His, exon 8

^c^ Significant for U.S. Foothills Ecoregion

^d^ Significant for U.S. High Plains Ecoregion

^e^ Significant for U.S. Arid Prairie Ecoregion

^f^ Significant for U.S. Southeast Ecoregion

^g^ Significant for U.S. Upper Midwest & Northeast Ecoregion

F

**Table S9.** Summary of GxE interactions with 2 to 4 supporting SNPs detected for yearling weight in U.S. Red Angus cattle.

| **Chr_Mb** | **-log_10_**  **P-value** | **Regression**  **Beta** | **MAF** | **Supporting**  **SNPs** | **Positional**  **Candidate**  **Genes** | **Lead SNP**  **Location** | **Scientific Precedence [reference]; organism; trait** |
| --- | --- | --- | --- | --- | --- | --- | --- |
| *8_15^a^* | 6.201 | 228.864 | 0.025 | 2 | *LOC112447774* | 3’ UTR | [2, 59]; Cattle; Milk fatty acid association, conception rate |
| *8_18^a^* | 6.156 | 207.614 | 0.024 | 2 | *LOC101904667, LOC112447778* | Intergenic | [1–3]; Cattle; mature weight, conception rate, body structure, calving ease |
| *12_50^a^* | 6.062 | 169.881 | 0.041 | 4 | *TBC1D4* | Intron | [3, 14, 40, 45, 65]; Human, cattle; Insulin and exercise response signaling, mediator of glucose uptake, conception rate, shear force, somatic cell score |
| *14_18^b^* | 6.045 | -9.269 | 0.431 | 4 | LOC100139328 | Intron | [3, 62, 66–69]; Cattle, mice; Milk fatty acid, conception rate, scrotal circumference, carcass weight, mitochondrial activity, metabolic homeostasis, and body weight |
| *24_06^a^* | 5.990 | -204.590 | 0.019 | 4 | *LOC112444205, LOC101907394* | Intergenic | [3, 43, 66, 70]; Cattle; Conception rate, milk fatty acid association |
| *24_07^a^* | 5.900 | 203.114 | 0.011 | 3 | *SOCS6, RTTN* | Intergenic | [14, 43, 66, 71, 72]; Mouse, human, cattle; Cell growth suppressor, embryonic development, response to insulin stimulation implicated in regulation of food intake and body weight, neuronal differentiation, brain development, severe growth failure, milk protein percentage and fatty acids, conception rate |
| *23_38^c^* | 5.672 | -10.683 | 0.348 | 2 | *MBOAT1* | Intron | [54, 73, 74]; Mouse, cattle; Neurite outgrowth and function, cell membrane biogenesis, mid-test metabolic weight test association |
| *11_30^c^* | 5.648 | -9.976 | 0.474 | 2 | *LOC112448871, TRNAC-GCA* | Intergenic | [7, 27, 40, 43]; Cattle; Conception rate, milk fat yield and percentage, milk protein percentage |
| *11_53^d^* | 5.563 | 11.977 | 0.133 | 2 | *LOC112448898, LOC101903989* | Intergenic | [40, 45, 75]; Cattle; Fatty acid in intramuscular fat, shear force, milk fatty acid, conception rate |
| *2_76^e^* | 5.386 | 10.244 | 0.485 | 3 | *LOC101902632, CNTNAP5* | Intergenic | [54, 76, 77]; Human, cattle; Regulator of synapse formation and neurotransmission, body length, hip cross height, mid-test metabolic weight test association |
| *27_39^f^* | 5.248 | 8.056 | 0.436 | 4 | *PSD3* | Intron | [2, 3, 7, 33, 38, 58–60]; Human, cattle; Obesity and type II diabetes, milk fat percentage, mature weight, body height, intramuscular fat, shear force, conception rate |
| *14_58^g^* | 5.509 | -18.835 | 0.401 | 4 | *OXR1* | Intron | [3, 46, 62, 78–80]; Human, mouse, cattle; Cell cycle regulation and apoptosis, protection of neuronal cells from oxidative stress, regulation of glucose metabolism in the brain, bicep weight, chuck roll weight, shear force, conception rate, scrotal circumference |
| *23_45^c^* | 5.317 | -9.576 | 0.478 | 2 | LOC112443738 | Intron | [31, 81]; Cattle; Fatty acid in muscle, milk fatty acid association |
| *12_43^g^* | 5.049 | -22.956 | 0.190 | 2 | *LOC107132995, KLHL1* | Intergenic | [2, 43, 82–86]; Human, rat, cattle, dog; Neurite outgrowth, milk yield and lactation persistence, sperm motility, conception rate, mature weight, yearling height, body weight |

^a^ Significant for U.S. Foothills Ecoregion

^b^ Significant for U.S. Fescue Belt Ecoregion

^c^ Significant for U.S. Upper Midwest & Northeast Ecoregion

^d^ Significant for U.S. High Plains Ecoregion

^e^ Significant for U.S. Forested Mountains Ecoregion

^f^ Significant for U.S. Arid Prairie Ecoregion

^g^ Significant for U.S. Southeast Ecoregion

**Additional File References**

1. Saatchi M, Schnabel RD, Taylor JF, Garrick DJ. Large-effect pleiotropic or closely linked QTL segregate within and across ten US cattle breeds. BMC Genomics. 2014;15.

2. Crispim AC, Kelly MJ, Guimarães SEF, e Silva FF, Fortes MRS, Wenceslau RR, et al. Multi-Trait GWAS and New Candidate Genes Annotation for Growth Curve Parameters in Brahman Cattle. PLoS One. 2015;10:e0139906. doi:10.1371/journal.pone.0139906.

3. Kiser JN, Keuter EM, Seabury CM, Neupane M, Moraes JGN, Dalton J, et al. Validation of 46 loci associated with female fertility traits in cattle. BMC Genomics. 2019;20:576. doi:10.1186/s12864-019-5935-3.

4. Veerkamp RF, Coffey MP, Berry DP, De Haas Y, Strandberg E, Bovenhuis H, et al. Genome-wide associations for feed utilisation complex in primiparous Holstein-Friesian dairy cows from experimental research herds in four European countries. Animal. 2012;6:1738–49. doi:10.1017/S1751731112001152.

5. Smith JL, Wilson ML, Nilson SM, Rowan TN, Oldeschulte DL, Schnabel RD, et al. Genome-wide association and genotype by environment interactions for growth traits in U.S. Gelbvieh cattle. BMC Genomics. 2019;20:926. doi:10.1186/s12864-019-6231-y.

6. Portela VM, Machado M, Buratini J, Zamberlam G, Amorim RL, Goncalves P, et al. Expression and Function of Fibroblast Growth Factor 18 in the Ovarian Follicle in Cattle1. Biol Reprod. 2010;83:339–46. doi:10.1095/biolreprod.110.084277.

7. Meredith BK, Kearney FJ, Finlay EK, Bradley DG, Fahey AG, Berry DP, et al. Genome-wide associations for milk production and somatic cell score in Holstein-Friesian cattle in Ireland. BMC Genet. 2012;13:21. doi:10.1186/1471-2156-13-21.

8. Fortes MRS, Reverter A, Nagaraj SH, Zhang Y, Jonsson NN, Barris W, et al. A single nucleotide polymorphism-derived regulatory gene network underlying puberty in 2 tropical breeds of beef cattle1. J Anim Sci. 2011;89:1669–83. doi:10.2527/jas.2010-3681.

9. Gasser CL, Behlke EJ, Grum DE, Day ML. Effect of timing of feeding a high-concentrate diet on growth and attainment of puberty in early-weaned heifers. J Anim Sci. 2006;84:3118–22. doi:10.2527/jas.2005-676.

10. Patel K, Scrimieri F, Ghosh S, Zhong J, Kim M-S, Ren YR, et al. FAM190A Deficiency Creates a Cell Division Defect. Am J Pathol. 2013;183:296–303. doi:10.1016/j.ajpath.2013.03.020.

11. Sebastian K, Detro-Dassen S, Rinis N, Fahrenkamp D, Müller-Newen G, Merk HF, et al. Characterization of SLCO5A1/OATP5A1, a Solute Carrier Transport Protein with Non-Classical Function. PLoS One. 2013;8:e83257. doi:10.1371/journal.pone.0083257.

12. Jang H, Choi Y, Yoo I, Han J, Kim M, Ka H. Expression and regulation of prostaglandin transporters, ATP-binding cassette, subfamily C, member 1 and 9, and solute carrier organic anion transporter family, member 2A1 and 5A1 in the uterine endometrium during the estrous cycle and pregnancy in pigs. Asian-Australasian J Anim Sci. 2016;30:643–52. doi:10.5713/ajas.16.0637.

13. Levy S, Allerston CK, Liveanu V, Habib MR, Gileadi O, Schuster G. Identification of LACTB2, a metallo-β-lactamase protein, as a human mitochondrial endoribonuclease. Nucleic Acids Res. 2016;44:1813–32.

14. Cole JB, Wiggans GR, Ma L, Sonstegard TS, Lawlor TJ, Crooker BA, et al. Genome-wide association analysis of thirty one production, health, reproduction and body conformation traits in contemporary U.S. Holstein cows. BMC Genomics. 2011;12:408. doi:10.1186/1471-2164-12-408.

15. Sanui T, Inayoshi A, Noda M, Iwata E, Oike M, Sasazuki T, et al. DOCK2 is essential for antigen-induced translocation of TCR and lipid rafts, but not PKC-θ and LFA-1, in T cells. Immunity. 2003;19:119–29.

16. Fukui Y, Hashimoto O, Sanui T, Oono T, Koga H, Abe M, et al. Haematopoietic cell-specific CDM family protein DOCK2 is essential for lymphocyte migration. Nature. 2001;412:826–31. doi:10.1038/35090591.

17. Espinosa-Diez C, Wilson R, Mukherjee R, Feltham M, Hudson C, Ruhl R, et al. DNA damage dependent hypomethylation regulates the pro-angiogenic LncRNA MEG9. bioRxiv. 2018;:442699. doi:10.1101/442699.

18. De Klerk B, Emam M, Thompson-Crispi KA, Sargolzaei M, Van der Poel JJ, Mallard BA. A genome-wide association study for natural antibodies measured in blood of Canadian Holstein cows. BMC Genomics. 2018;19:694. doi:10.1186/s12864-018-5062-6.

19. Akanno EC, Chen L, Abo-Ismail MK, Crowley JJ, Wang Z, Li C, et al. Genome-wide association scan for heterotic quantitative trait loci in multi-breed and crossbred beef cattle. Genet Sel Evol. 2018;50:48. doi:10.1186/s12711-018-0405-y.

20. Urano T, Shiraki M, Yagi H, Ito M, Sasaki N, Sato M, et al. *GPR98* / *Gpr98* Gene Is Involved in the Regulation of Human and Mouse Bone Mineral Density. J Clin Endocrinol Metab. 2012;97:E565–74. doi:10.1210/jc.2011-2393.

21. Zhang F, Wang Y, Mukiibi R, Chen L, Vinsky M, Plastow G, et al. Genetic architecture of quantitative traits in beef cattle revealed by genome wide association studies of imputed whole genome sequence variants: I: feed efficiency and component traits. BMC Genomics. 2020;21:36. doi:10.1186/s12864-019-6362-1.

22. Hu W, Zuo J, De Jager PL, Heintz N. The human glutamate receptor δ2 gene (GRID2) maps to chromosome 4q22. Genomics. 1998;47:143–5.

23. Stafuzza NB, Costa e Silva EV da, Silva RM de O, Costa Filho LCC da, Barbosa FB, Macedo GG, et al. Genome‐wide association study for age at puberty in young Nelore bulls. J Anim Breed Genet. 2020;137:234–44. doi:10.1111/jbg.12438.

24. Howard JT, Kachman SD, Snelling WM, Pollak EJ, Ciobanu DC, Kuehn LA, et al. Beef cattle body temperature during climatic stress: A genome-wide association study. Int J Biometeorol. 2014;58:1665–72. doi:10.1007/s00484-013-0773-5.

25. Franzoni E, Booker SA, Parthasarathy S, Rehfeld F, Grosser S, Srivatsa S, et al. miR-128 regulates neuronal migration, outgrowth and intrinsic excitability via the intellectual disability gene Phf6. Elife. 2015;4.

26. Palmer ND, McDonough CW, Hicks PJ, Roh BH, Wing MR, An SS, et al. A genome-wide association search for type 2 diabetes genes in african americans. PLoS One. 2012;7. doi:10.1371/journal.pone.0029202.

27. Marete AG, Guldbrandtsen B, Lund MS, Fritz S, Sahana G, Boichard D. A Meta-Analysis Including Pre-selected Sequence Variants Associated With Seven Traits in Three French Dairy Cattle Populations. Front Genet. 2018;9:522. doi:10.3389/fgene.2018.00522.

28. Anitha A, Thanseem I, Nakamura K, Vasu MM, Yamada K, Ueki T, et al. Zinc finger protein 804A (ZNF804A) and verbal deficits in individuals with autism. J Psychiatry Neurosci. 2014;39:294–303. doi:10.1503/jpn.130126.

29. Doyle JL, Berry DP, Veerkamp RF, Carthy TR, Evans RD, Walsh SW, et al. Genomic regions associated with muscularity in beef cattle differ in five contrasting cattle breeds. Genet Sel Evol. 2020;52:1–18. doi:10.1186/s12711-020-0523-1.

30. Leal-Gutiérrez JD, Elzo MA, Mateescu RG. Identification of eQTLs and sQTLs associated with meat quality in beef. BMC Genomics. 2020;21:1–15. doi:10.1186/s12864-020-6520-5.

31. Lemos MVA, Chiaia HLJ, Berton MP, Feitosa FLB, Aboujaoud C, Camargo GMF, et al. Genome-wide association between single nucleotide polymorphisms with beef fatty acid profile in Nellore cattle using the single step procedure. BMC Genomics. 2016;17:213. doi:10.1186/s12864-016-2511-y.

32. Calder MD, Madan P, Watson AJ. Bovine oocytes and early embryos express Staufen and ELAVL RNA-binding proteins. Zygote. 2008;16:161–8. doi:10.1017/S096719940700456X.

33. Bouwman AC, Bovenhuis H, Visker MHPW, van Arendonk JAM. Genome-wide association of milk fatty acids in Dutch dairy cattle. BMC Genet. 2011;12:43. doi:10.1186/1471-2156-12-43.

34. Berto S, Usui N, Konopka G, Fogel BL. ELAVL2-regulated transcriptional and splicing networks in human neurons link neurodevelopment and autism. Hum Mol Genet. 2016;25:2451–64. doi:10.1093/hmg/ddw110.

35. Heni M, Kullmann S, Ahlqvist E, Wagner R, Machicao F, Staiger H, et al. Interaction between the obesity-risk gene FTO and the dopamine D2 receptor gene ANKK1/TaqIA on insulin sensitivity. Diabetologia. 2016;59:2622–31. doi:10.1007/s00125-016-4095-0.

36. Rubio-Solsona E, Marti S, Vílchez JJ, Palau F, Hoenicka J. ANKK1 is found in myogenic precursors and muscle fibers subtypes with glycolytic metabolism. PLoS One. 2018;13. doi:10.1371/journal.pone.0197254.

37. Bouwman AC, Daetwyler HD, Chamberlain AJ, Ponce CH, Sargolzaei M, Schenkel FS, et al. Meta-analysis of genome-wide association studies for cattle stature identifies common genes that regulate body size in mammals. Nat Genet. 2018;50:362–7. doi:10.1038/s41588-018-0056-5.

38. Yan Z, Wang Z, Zhang Q, Yue S, Yin B, Jiang Y, et al. Identification of whole‐genome significant single nucleotide polymorphisms in candidate genes associated with body conformation traits in Chinese Holstein cattle. Anim Genet. 2020;51:141–6. doi:10.1111/age.12865.

39. Oliveira HR, Cant JP, Brito LF, Feitosa FLB, Chud TCS, Fonseca PAS, et al. Genome-wide association for milk production traits and somatic cell score in different lactation stages of Ayrshire, Holstein, and Jersey dairy cattle. J Dairy Sci. 2019;102:8159–74. doi:10.3168/jds.2019-16451.

40. Höglund JK, Sahana G, Guldbrandtsen B, Lund MS. Validation of associations for female fertility traits in Nordic Holstein, Nordic Red and Jersey dairy cattle. BMC Genet. 2014;15:8. doi:10.1186/1471-2156-15-8.

41. Theurey P, Rieusset J. Mitochondria-Associated Membranes Response to Nutrient Availability and Role in Metabolic Diseases. Trends in Endocrinology and Metabolism. 2017;28:32–45.

42. Rieusset J. The role of endoplasmic reticulum-mitochondria contact sites in the control of glucose homeostasis: An update. Cell Death Dis. 2018;9:1–12. doi:10.1038/s41419-018-0416-1.

43. Galliou JM, Kiser JN, Oliver KF, Seabury CM, Moraes JGN, Burns GW, et al. Identification of Loci and Pathways Associated with Heifer Conception Rate in U.S. Holsteins. Genes (Basel). 2020;11:767. doi:10.3390/genes11070767.

44. Hansen L, Comyn S, Mang Y, Lind-Thomsen A, Myhre L, Jean F, et al. The myosin chaperone UNC45B is involved in lens development and autosomal dominant juvenile cataract. Eur J Hum Genet. 2014;22:1290–7. doi:10.1038/ejhg.2014.21.

45. McClure MC, Ramey HR, Rolf MM, McKay SD, Decker JE, Chapple RH, et al. Genome‐wide association analysis for quantitative trait loci influencing Warner–Bratzler shear force in five taurine cattle breeds. Anim Genet. 2012;43:662–73. doi:10.1111/j.1365-2052.2012.02323.x.

46. Mateescu RG, Garrick DJ, Reecy JM. Network analysis reveals putative genes affecting meat quality in Angus cattle. Front Genet. 2017;8 NOV. doi:10.3389/fgene.2017.00171.

47. Dikmen S, Wang X -z., Ortega MS, Cole JB, Null DJ, Hansen PJ. Single nucleotide polymorphisms associated with thermoregulation in lactating dairy cows exposed to heat stress. J Anim Breed Genet. 2015;132:409–19. doi:10.1111/jbg.12176.

48. Ortega MS, Denicol AC, Cole JB, Null DJ, Hansen PJ. Use of single nucleotide polymorphisms in candidate genes associated with daughter pregnancy rate for prediction of genetic merit for reproduction in Holstein cows. Anim Genet. 2016;47:288–97. doi:10.1111/age.12420.

49. Barendse W. Haplotype analysis improved evidence for candidate genes for intramuscular fat percentage from a genome wide association study of cattle. PLoS One. 2011;6. doi:10.1371/journal.pone.0029601.

50. Karisa BK, Thomson J, Wang Z, Stothard P, Moore SS, Plastow GS. Candidate genes and single nucleotide polymorphisms associated with variation in residual feed intake in beef cattle. J Anim Sci. 2013;91:3502–13. doi:10.2527/jas.2012-6170.

51. Nelovkov A, Philips MA, Kõks S, Vasar E. Rats with low exploratory activity in the elevated plus-maze have the increased expression of limbic system-associated membrane protein gene in the periaqueductal grey. Neurosci Lett. 2003;352:179–82.

52. Pimenta AF, Zhukareva V, Barbe MF, Reinoso BS, Grimley C, Henzel W, et al. The limbic system-associated membrane protein is an Ig superfamily member that mediates selective neuronal growth and axon targeting. Neuron. 1995;15:287–97. doi:10.1016/0896-6273(95)90034-9.

53. Pszczola M, Strabel T, Mucha S, Sell-Kubiak E. Genome-wide association identifies methane production level relation to genetic control of digestive tract development in dairy cows. Sci Rep. 2018;8:15164. doi:10.1038/s41598-018-33327-9.

54. Lu D, Miller S, Sargolzaei M, Kelly M, Vander Voort G, Caldwell T, et al. Genome-wide association analyses for growth and feed efficiency traits in beef cattle. J Anim Sci. 2013;91:3612–33. doi:10.2527/jas.2012-5716.

55. Howard JM, Nuguid JM, Ngole D, Nguyen H. Tcf3 expression marks both stem and progenitor cells in multiple epithelia. Dev. 2014;141:3143–52. doi:10.1242/dev.106989.

56. Lau LY, Nguyen LT, Reverter A, Moore SS, Lynn A, McBride-Kelly L, et al. Gene regulation could be attributed to TCF3 and other key transcription factors in the muscle of pubertal heifers. Vet Med Sci. 2020;6:695–710. doi:10.1002/vms3.278.

57. Liu W, Liu Y, Guo T, Hu C, Luo H, Zhang L, et al. TCF3, a novel positive regulator of osteogenesis, plays a crucial role in miR-17 modulating the diverse effect of canonical Wnt signaling in different microenvironments. Cell Death Dis. 2013;4:539. doi:10.1038/cddis.2013.65.

58. Gong S, Xu C, Wang L, Liu Y, Owusu D, Bailey BA, et al. Genetic association analysis of polymorphisms in PSD3 gene with obesity, type 2 diabetes, and HDL cholesterol. Diabetes Res Clin Pract. 2017;126:105–14.

59. Ibeagha-Awemu EM, Peters SO, Akwanji KA, Imumorin IG, Zhao X. High density genome wide genotyping-by-sequencing and association identifies common and low frequency SNPs, and novel candidate genes influencing cow milk traits. Sci Rep. 2016;6:1–18. doi:10.1038/srep31109.

60. Frischknecht M, Seefried FR, Signer-Hasler H, Garrick D, Stricker C, Consortium I, et al. Genome-wide association studies of fertility and calving traits in Brown Swiss cattle using imputed whole-genome sequences. BMC Genomics. 2017;18:1–13. doi:10.1186/s12864-017-4308-z.

61. Zhou Y, Connor EE, Wiggans GR, Lu Y, Tempelman RJ, Schroeder SG, et al. Genome-wide copy number variant analysis reveals variants associated with 10 diverse production traits in Holstein cattle. BMC Genomics. 2018;19:314. doi:10.1186/s12864-018-4699-5.

62. Buzanskas ME, Grossi D do A, Ventura RV, Schenkel FS, Chud TCS, Stafuzza NB, et al. Candidate genes for male and female reproductive traits in Canchim beef cattle. J Anim Sci Biotechnol. 2017;8:67. doi:10.1186/s40104-017-0199-8.

63. Sahana G, Guldbrandtsen B, Lund MS. Genome-wide association study for calving traits in Danish and Swedish Holstein cattle. J Dairy Sci. 2011;94:479–86.

64. Hardie LC, VandeHaar MJ, Tempelman RJ, Weigel KA, Armentano LE, Wiggans GR, et al. The genetic and biological basis of feed efficiency in mid-lactation Holstein dairy cows. J Dairy Sci. 2017;100:9061–75. doi:10.3168/jds.2017-12604.

65. Moltke I, Grarup N, Jørgensen ME, Bjerregaard P, Treebak JT, Fumagalli M, et al. A common Greenlandic TBC1D4 variant confers muscle insulin resistance and type 2 diabetes. Nature. 2014;512:190–3. doi:10.1038/nature13425.

66. Gebreyesus G, Buitenhuis AJ, Poulsen NA, Visker MHPW, Zhang Q, van Valenberg HJF, et al. Combining multi-population datasets for joint genome-wide association and meta-analyses: The case of bovine milk fat composition traits. J Dairy Sci. 2019;102:11124–41.

67. Srikanth K, Lee SH, Chung KY, Park JE, Jang GW, Park MR, et al. A gene-set enrichment and protein–protein interaction network-based gwas with regulatory snps identifies candidate genes and pathways associated with carcass traits in hanwoo cattle. Genes (Basel). 2020;11:316. doi:10.3390/genes11030316.

68. Júnior GAF, Costa RB, de Camargo GMF, Carvalheiro R, Rosa GJM, Baldi F, et al. Genome scan for postmortem carcass traits in Nellore cattle. J Anim Sci. 2016;94:4087–95. doi:10.2527/jas2016-0632.

69. Quirós PM, Ramsay AJ, Sala D, Fernández-Vizarra E, Rodríguez F, Peinado JR, et al. Loss of mitochondrial protease OMA1 alters processing of the GTPase OPA1 and causes obesity and defective thermogenesis in mice. EMBO J. 2012;31:2117–33. doi:10.1038/emboj.2012.70.

70. Gebreyesus G, Buitenhuis AJ, Poulsen NA, Visker MHPW, Zhang Q, Van Valenberg HJF, et al. Multi-population GWAS and enrichment analyses reveal novel genomic regions and promising candidate genes underlying bovine milk fatty acid composition. BMC Genomics. 2019;20:178. doi:10.1186/s12864-019-5573-9.

71. Krebs DL, Uren RT, Metcalf D, Rakar S, Zhang J-G, Starr R, et al. SOCS-6 Binds to Insulin Receptor Substrate 4, and Mice Lacking the SOCS-6 Gene Exhibit Mild Growth Retardation. Mol Cell Biol. 2002;22:4567–78. doi:10.1128/mcb.22.13.4567-4578.2002.

72. Grandone A, Torella A, Santoro C, Giugliano T, del Vecchio Blanco F, Mutarelli M, et al. Expanding the phenotype of *RTTN* variations: a new family with primary microcephaly, severe growth failure, brain malformations and dermatitis. Clin Genet. 2016;90:445–50. doi:10.1111/cge.12771.

73. Tabe S, Hikiji H, Ariyoshi W, Hashidate-Yoshida T, Shindou H, Okinaga T, et al. Lysophosphatidylethanolamine acyltransferase 1/membrane-bound O-acyltransferase 1 regulates morphology and function of P19C6 cell-derived neurons. FASEB J. 2016;30:2591–601. doi:10.1096/fj.201500097R.

74. Hishikawa D, Shindou H, Kobayashi S, Nakanishi H, Taguchi R, Shimizu T. Discovery of a lysophospholipid acyltransferase family essential for membrane asymmetry and diversity. Proc Natl Acad Sci U S A. 2008;105:2830–5. doi:10.1073/pnas.0712245105.

75. Vinicius M, De Lemos A, Peripolli E, Piatto Berton M, Loise F, Feitosa B, et al. Association study between copy number variation and beef fatty acid profile of Nellore cattle. J Appl Genet. 2018;59:203–23. doi:10.1007/s13353-018-0436-7.

76. Vanvanhossou SFU, Scheper C, Dossa LH, Yin T, Brügemann K, König S. A multi-breed GWAS for morphometric traits in four Beninese indigenous cattle breeds reveals loci associated with conformation, carcass and adaptive traits. BMC Genomics. 2020;21. doi:10.1186/s12864-020-07170-0.

77. Chen Q, Huang B, Zhan J, Wang J, Qu K, Zhang F, et al. Whole-genome analyses identify loci and selective signals associated with body size in cattle. J Anim Sci. 2020;98:1–8. doi:10.1093/jas/skaa068.

78. Yang M, Lin X, Rowe A, Rognes T, Eide L, Bjørås M. Transcriptome analysis of human OXR1 depleted cells reveals its role in regulating the p53 signaling pathway. Sci Rep. 2015;5:17409. doi:10.1038/srep17409.

79. Oliver PL, Finelli MJ, Edwards B, Bitoun E, Butts DL, Becker EBE, et al. Oxr1 Is Essential for Protection against Oxidative Stress-Induced Neurodegeneration. PLoS Genet. 2011;7:e1002338. doi:10.1371/journal.pgen.1002338.

80. Zhang R, Miao J, Song Y, Zhang W, Xu L, Chen Y, et al. Genome-wide association study identifies the PLAG1-OXR1 region on BTA14 for carcass meat yield in cattle. Physiol Genomics. 2019;51:137–44. doi:10.1152/physiolgenomics.00112.2018.-Car.

81. Bouwman AC, Visker MHPW, van Arendonk JAM, Bovenhuis H. Genomic regions associated with bovine milk fatty acids in both summer and winter milk samples. BMC Genet. 2012;13:93. doi:10.1186/1471-2156-13-93.

82. Seng S, Avraham HK, Jiang S, Venkatesh S, Avraham S. KLHL1/MRP2 Mediates Neurite Outgrowth in a Glycogen Synthase Kinase 3β-Dependent Manner. Mol Cell Biol. 2006;26:8371–84. doi:10.1128/mcb.02167-05.

83. Yue SJ, Zhao YQ, Gu XR, Yin B, Jiang YL, Wang ZH, et al. A genome-wide association study suggests new candidate genes for milk production traits in Chinese Holstein cattle. Anim Genet. 2017;48:677–81. doi:10.1111/age.12593.

84. Hering DM, Olenski K, Kaminski S. Genome-wide association study for poor sperm motility in Holstein-Friesian bulls. Anim Reprod Sci. 2014;146:89–97.

85. Terakado APN, Costa RB, De Camargo GMF, Irano N, Bresolin T, Takada L, et al. Genome-wide association study for growth traits in Nelore cattle. Animal. 2018;12:1358–62. doi:10.1017/S1751731117003068.

86. Sheet S, Krishnamoorthy S, Cha J, Choi S, Choi B-H. Identification of Candidate Genes and Pathways Associated with Obesity-Related Traits in Canines via Gene-Set Enrichment and Pathway-Based GWAS Analysis. Animals. 2020;10:1–15.
